# Identification of A Novel Antioxidant Tripeptide Ameliorating Skin Photoaging

**DOI:** 10.1111/jocd.70977

**Published:** 2026-06-10

**Authors:** Jianghua Lu, Jie Li, Liang wang, Liming Xia, Yang Wan, Qingyong Li

**Affiliations:** ^1^ Guangzhou Yiyang Biotechnology Co., Ltd Guangzhou China; ^2^ Guangzhou Jnumeso Bio‐technology Co., Ltd Guangzhou China

**Keywords:** FAW, oxidative stress, photoaging, photodamage, skin

## Abstract

**Background:**

Skin photodamage induced by ultraviolet (UV) exposure is the primary factor of photoaging, and developing bioactive molecules targeting this process represents a key focus in dermatology and cosmetic biotechnology. However, a comprehensive screening strategy combining combinatorial library construction with multi‐target validation has not yet been fully established.

**Objective:**

To establish a systematic screening strategy for bioactive peptide molecules and to assess the antioxidant, anti‐photoaging effects of the identified bioactive peptide on skin cells.

**Methods:**

In this study, 17 out of 20 common amino acids were chosen according to their antioxidant capacity via H_2_O_2_‐ and UVB‐induced oxidative stress models. Based on these 17 amino acids, a tripeptide library containing 4913 molecules was constructed using a full combinatorial strategy. With skin melanin synthesis (tyrosinase), extracellular matrix degradation (elastase or hyaluronidase), and oxidative stress regulation (Keap1) as core targets, 10 potential bioactive tripeptides were screened out through molecular docking technology. Subsequently, mitochondrial function was examined by detecting mitochondrial membrane potential and mitochondrial superoxide anion levels to evaluate the antioxidant activity of the identified tripeptide. Detection of mRNA levels of antioxidant‐related genes and RNA‐seq analysis were performed to investigate the associated signaling pathways. Additionally, senescence‐associated β‐galactosidase staining was used to assess the anti‐aging capacity.

**Results:**

In vitro functional validation confirmed that tripeptide FAW exhibited the optimal performance in migration capacity and antioxidant activity for skin cells. Mechanistic studies revealed that FAW may enhance cellular antioxidant capacity by activating the NRF2/HMOX‐1 pathway and regulate skin aging‐related signaling pathways. The results showed that FAW could alleviated UV‐induced skin cell damage and photoaging, and ameliorate passage number‐induced replicative senescence.

**Conclusion:**

This study established a screening strategy for bioactive peptide molecules, encompassing amino acid screening, tripeptide library construction, multi‐target molecular docking, and functional validation. Notably, tripeptide FAW identified in this study showed promising application potential for skincare.

## 1 Introduction

Skin aging is a complex process, which is induced by intrinsic and extrinsic factors. Intrinsic aging is mainly due to genetic and chronological factors, while extrinsic aging, especially photoaging, is significantly affected by environmental factors such as ultraviolet (UV) radiation. UV radiation is a main cause of photoaging. UVA (320–400 nm) reaching the basal and dermal layers, induces ROS, activates the mitogen‐activated protein kinase pathway, promotes matrix metalloproteinases expression, and inhibits collagen synthesis, leading to wrinkles, laxity, and pigmentation [1]. UVB (280–320 nm), acting on the epidermis, causes immediate damage like erythema and longterm effects such as increased MMPs expression and reduced collagen production [2, 3]. Research also focuses on the immune system's role in photoaging. UV‐induced oxidative stress disrupts immune cells like Langerhans cells, weakening the body's defense and promoting skin photoaging [4, 5, 6]. Additionally, air pollutants like particulate matter, polycyclic aromatic hydrocarbons, and heavy metals interact with UV, generating ROS in the skin and accelerating skin aging [7, 8].

Recent studies have identified that Trp, Met, His, Lys, Cys, Arg and Tyr were identified as antioxidant amino acids by the micro‐potassium permanganate method [9]. Furthermore, ABTs radical‐scavenging activity assay demonstrated that Cys, Trp, Tyr, Arg, His, Lys, Met, Phe, Val amino acids had antioxidant capacity. The results of DPPH radical‐scavenging assay and Ferric reducing antioxidant power assay showed that Cys was the only amino acid with antioxidant effect among the 20 amino acids [10]. Despite these findings, inconsistencies between biochemical assays and in vitro cellular assays have been observed. The limited research on the cellular antioxidant activity of amino acids highlights a need for further investigation in this area.

We aimed to find tripeptides with amino acids that have antioxidant effects. Tripeptides offer distinct advantages over dipeptides, tetrapeptides, and longer polypeptides. Compared to dipeptides, their trimeric structure allows for greater functional diversity, while their shorter chain length relative to longer polypeptides enhances stability, reduces synthesis costs, and improves transdermal absorption and enzymatic resistance [11]. These properties enable tripeptides to exhibit high cost‐effectiveness and broad applications in pharmaceuticals, cosmetics, and food industries.

Hyaluronidase, elastase and tyrosinase compositely affect skin homeostasis: hyaluronidase and elastase control the integrity of the extracellular matrix [12, 13], while tyrosinase regulates pigmentation [14]. Their dysregulation of activity can lead to aging, inflammation and pigmentation disorders, making them key targets in dermatological research and treatment development. Peptides as active ingredients, characterized by high nutritional value, good bioavailability, low or no immune response, have been paid more attention. So far, many peptides with antioxidant activity have been reported. However, it's still unclear which peptide with antioxidant properties has anti‐skin aging ability. On the one hand, cosmetic ingredients with effective antioxidant activity are important in mitigating skin aging. On the other hand, the moisturizing and barrier repair function are essential for maintaining skin homeostasis. Therefore, for peptide exerting anti‐skin aging, one crucial strategy is to explore peptides with antioxidant, moisturizing and barrier repair function. Therefore, it is very important to develop tripeptides that can inhibit hyaluronidase, elastase and tyrosinase, and have good antioxidant capacity, which can delay skin aging [15].

The current research focused on countering photoaging was on effective antioxidants. As we known, oxidative stress from excessive ROS during photoaging will disrupts normal cell function. Nuclear factor erythroid 2‐related factor 2 (NRF2) signaling pathway is a key regulator in the cell response to oxidative stress. In this process, the transcription factor NRF2 can activate antioxidant response element (ARE)‐mediated gene expression. In skin cells, NRF2 activation enhances endogenous anti‐oxidase production, neutralizing ROS and maintaining redox balance, thus protecting against photoaging [16, 17, 18]. Therefore, for ameliorating photoaging, much attention should be focused on the antioxidant that can activate NRF2 signaling pathway in addition to reducing ROS.

In this study, 17 amino acids with antioxidant capacity for skin cells were first screened out. Using molecular docking technology, a 4913 tripeptides library randomly composed of 17 amino acids with antioxidant activity was screened for their binding capabilities with hyaluronidase, elastase, tyrosinase, and Keap1‐Kelch domain receptor. Subsequently, these potential anti‐aging peptides were further verified through a series of in vitro experiments, including enzyme activity inhibition tests, cell viability, antioxidant activity, and migration ability tests, and the promising tripeptide FAW was selected. Through RNA‐Sequence analysis, we explored the potential mechanism of anti‐aging peptides. Finally, FAW has antioxidant activity, which is beneficial for improving skin photoaging and exerts a positive effect on skincare.

## 2 Materials and Methods

### 2.1 Cell Culture

The human immortalized keratinocyte cells (HaCaT), which were present in this study, were obtained from the Conservation Genetics CAS Kunming Cell Bank (CHN) and cultured in DMEM medium (Gibco, USA) supplemented with 10% fetal bovine serum (FBS, Gibco, USA) in a 37°C incubator containing 5% CO_2_. The human skin fibroblasts (HSF), which were present in this study, were obtained from the Beijing EallBio Biomedical Technology Co. Ltd. and cultured in DMEM/F12 medium (Gibco, USA) supplemented with 10% fetal bovine serum (FBS, Gibco, USA) in a 37°C incubator containing 5% CO_2_.

### 2.2 UV Irradiation Treatment

HaCaT was cultured in plates at a cell density of 3 × 10^4^ cells/cm^2^ and were incubated in a cell culture incubator for 24 h. HaCaT were exposed to UVB radiation using a TL 20 W/01 lamp (PHILIPS, Belgium) with an irradiance 300 mJ/cm^2^. After the irradiation, the cells were cultured for an additional 24 h. To establish a photoaging model, HaCaT were irradiated with UVB three times at a radiation dose of 20 mJ/cm^2^ once a day. After the irradiation, the cells were cultured for an additional 72 h. HSF was cultured in plates at a cell density of 4.5 × 10^4^ cells/cm^2^ and were incubated in a cell culture incubator for 24 h. HSF was exposed to UVA radiation using a TL‐K 40 W/10R (PHILIPS, Belgium) with an irradiance 1500 mJ/cm^2^. After the irradiation, the cells were cultured for an additional 48 h. The intensity of the UVA/UVB radiation was measured using a UV radiometer (UV‐340A, Lutron, TaiWan) before and after irradiation. The control group was treated to the same conditions, but without any UV exposure or samples treatment.

### 2.3 Cell Viability Assay

The cytotoxic effects of amino acids, peptides, UVB irradiation on HaCaT or UVA irradiation on HSF were determined by using Cell Counting Kit‐8 (CCK‐8) (Beyotime, Shanghai, China). For CCK‐8 assays, HaCaT was seeded on a 96‐well plate and irradiated with or without UVB and treated with amino acids or peptides at different concentrations for 24 h. HSF was seeded on a 96‐well plate and irradiated with or without UVA and treated with FAW at different concentrations for 48 h. Cell viability rates were subsequently assessed using the CCK‐8 according to the manufacturer's instructions.

### 2.4 Flow Cytometry Assay

ROS production was measured using 2′,7′‐dichlorofluoresce in diacetate (H_2_DCFDA) (Sigma, D6883‐50MG) protocol. In brief, the cells were seeded on a 12‐well plate. After treatment with UVA or UVB or FAW on the cell, H_2_DCFDA (10 μmol/L) was diluted in basic medium and added to the cells. For the H_2_O_2_‐induced oxidative stress model, the medium containing H_2_DCFDA was kept at 37°C in a CO_2_ incubator for 45 min and then loaded for 30 min with or without H_2_O_2_ at different concentrations. Subsequently, ROS levels were measured by BD FACSCalibur Flow Cytometer [19].

### 2.5 Molecular Docking

4913 tripeptides, randomly composed of 17 antioxidant amino acids, were constructed to establish a ligand peptide library. Subsequently, we employed the Autodock Vina program to perform molecular docking studies on the tyrosinase, elastase, hyaluronidase, and Keap1‐Kelch domain according to the methods as described elsewhere with slight modification [20, 21]. Combining the antioxidant properties of the amino acid residues, we conducted a preliminary structure–activity relationship analysis based on the molecular docking results and selected several potential peptides with antioxidant activity. In this study, Autodock Vina scores were recorded, and the output results were visualized in 2D diagram through Discovery studio 2016 (DS) software for display of their mode of interaction with binding site residues.

### 2.6 Elastase Activity Inhibition Assay

Elastase (0.2 U/mL) and substrate N‐Succinyl‐Alanine‐Alanine‐Alanine‐p‐Nitroaniline (AAAPAN) (5.0 mmol/L) were prepared in 0.2 mol/L Tris–HCl buffer (pH 8.0). For the assay, 90 μL buffer mixed with 30 μL AAAPAN was incubated with 50 μL sample/control at 25°C for 15 min, then 30 μL elastase was added. After 15 min incubation, absorbance at 410 nm was measured. The inhibition rate was calculated as [1–(C–D)/(A–B)] × 100, where A, B, C, and D represent blank, negative control, sample, and sample blank groups, respectively.

### 2.7 Hyaluronidase Activity Inhibition Assay

Hyaluronidase (30 mg/mL) and hyaluronic acid (HA, 3 mg/mL) were prepared in acetate buffer (pH 3.5). For the assay, 50 μL hyaluronidase was preincubated with 50 μL sample at 37°C for 20 min, followed by 100 μL CaCl_2_ and 250 μL HA, each incubated for 20 min and 40 min, respectively. Reaction was terminated with NaOH and potassium borate, boiled for 3 min, ice‐cooled, and mixed with 3 mL p‐dimethylaminobenzaldehyde. Absorbance at 540 nm was measured after 20 min color development, with centrifugation (6000 rpm, 5 min) for precipitated samples. Inhibition rate was calculated as [1–(C–D)/(A–B)] × 100, where A, B, C, and D represent control, blank, sample, and sample control groups, respectively.

### 2.8 Detection of Hyaluronic Acid and Elastin Content

HSF were cultured to logarithmic growth phase, the cells were resuspended in complete medium, and the cell concentration was adjusted to 9.4 × 10^4^ cells/mL. Then, 1 mL of cell solution was added to each of the 24‐well plates, and the cells were cultured in an incubator at 37°C CO_2_ overnight until the confluence of the cells was complete. The control group was added complete medium, the UVA model group was exposed to UVA radiation using a TL‐K 40 W/10R (PHILIPS, Belgium), and the UVA plus sample groups were exposed to UVA radiation and treated with peptides of 100 μmol/L concentrations; the cells were cultured for 48 h. Cell culture supernatants were aspirated and set aside for subsequent assays. A HA assay kit (SEKH‐0509, Solarbio, China) and Elastin assay kit (MM‐1149H1, MEIMIAN, China) were used to detect the HA and elastin content in the cell culture supernatants, respectively. The final level of HA and elastin was then normalized to its protein content determined by a BCA protein assay kit (P0010, Beyotime).

### 2.9 Tyrosinase Activity Inhibition Assay

Tyrosinase (110 U/mL) was prepared in phosphate buffer and stored at −80°C. L‐3,4‐Dihydroxyphenylalanine (L‐DOPA) (1 mg/mL) was dissolved in the same buffer via sonication (avoiding prolonged sonication to prevent blackening) and protected from light. Test samples were dissolved in the buffer. In a 96‐well plate, 50 μL of sample was mixed with 150 μL L‐DOPA, followed by 50 μL tyrosinase. Absorbance at 475 nm was measured at 10 s (A1) and after 20 min incubation at 37°C (A2). The inhibition rate was calculated as [1–(A2_sample–A1_sample)/(A2_control–A1_control)] × 100%.

### 2.10 Determination of Cellular Melanin Content

Cellular melanin content was determined spectrophotometrically. Following treatment, adherent B16F10 cells were washed with PBS and harvested via trypsinization. After centrifugation, the pellets were lysed with 250 μL of M‐Lysis buffer (1 M NaOH containing 10% DMSO). The mixtures were incubated at 80°C for 15 min, cooled on ice, and centrifuged. The supernatants were transferred to a 96‐well plate, and absorbance was measured at 405 nm. Protein concentrations were determined by BCA assay at 562 nm for normalization. Results were calculated as relative melanin content, with M‐Lysis serving as the background blank.

### 2.11 Cell Migration Assay

HaCaT were seeded in 12‐well plates at 3 × 10^5^ cells/well and incubated for 24 h until 90% confluent. A scratch was made in the cell monolayer with a 200 μL pipette tip. After washing with phosphate‐buffered saline (PBS) to remove debris, DMEM was added to start the migration assay. Images of the scratch area were taken at 0 h and 24 h using an inverted microscope. The cell migration was quantified by measuring the recovered scratch area.

### 2.12 Measurement of the Mitochondrial Membrane Potential

Mitochondrial membrane potential was evaluated using the JC‐1 mitochondrial membrane potential assay kit (Beyotime Institute of Biotechnology, China). Following the manufacturer's protocols, fluorescence signals of JC‐1 monomers (green fluorescence) and aggregates (red fluorescence) were captured under a fluorescence microscope (Nikon). Digital images were processed and analyzed using ImageJ software, and the red‐to‐green fluorescence intensity ratio was calculated to quantify mitochondrial membrane potential.

### 2.13 Detection of Mitochondrial Reactive Oxygen Species

HaCaT were seeded at a density of 2 × 10^5^ cells/mL and subjected to UVB irradiation and FAW treatment. After treatment, the cells were rinsed with PBS and incubated with 5 μmol/L MitoSOX Red. Fluorescent images were captured using a Nikon imaging system.

### 2.14 Real‐Time Quantitative Polymerase Chain Reaction Analysis

At the end of the treatment, cells were collected. The entire RNA was extracted using HiPure Total RNA Mini Kit. Post quality checks and purification, 1 μg of the RNA was reverse transcribed to cDNA using a reverse transcription kit. Real‐time quantitative polymerase chain reaction analysis (RT‐qPCR) was performed using the TB Green Premix Ex Taq II (Tli RNaseH Plus) in a LightCycler 480II (Roche) detection system. GADPH was used as an internal reference, and the relative gene expression was calculated using the 2^−△△Ct^ method. The primer sequences used are listed in Table S2.

### 2.15 Senescence‐Associated β‐Galactosidase Activity

Aged HaCaT were stained using the senescence‐associated β‐galactosidase (SA‐β‐Gal) activity staining kit. 72 h after the last UVB irradiation, the cell culture medium was removed and washed with PBS. Then, 100 μL of fixative solution was added, and the cells were fixed at room temperature for 15 min. Subsequently, the samples were washed three times with PBS for 3 min each time. After removing the PBS, 100 μL of staining solution was added to each well, and the plates were placed in a humidified chamber at 37°C overnight. The morphology and staining of the cells were observed under an optical microscope.

### 2.16 RNA‐Seq Method

Following RNA extraction with TRIzol (15596026CN, Thermo), three groups of repeated HaCaT were used for RNA sequencing after treatment with FAW. This sequencing was completed by APT (APPLIED PROTEIN TECHNOLOGY). The data generated from Illumina (or BGI) platform were used for bio‐informatics analysis. All of the analyses were performed using an in‐house pipeline from Shanghai Applied Protein Technology. The genes with differential expression were screened using the DESeq2, with the following criterion: *p <* 0.05 and |log_2_fold change| > 0.585.

### 2.17 Statistical Analysis

All data were expressed as mean ± standard deviation (SD). The statistical analysis was conducted by using GraphPad Prism version 8.0.2. The significant difference was assessed by unpaired *t*‐test or one‐way analysis of variance (ANOVA). *p*‐values < 0.05 were considered to be statistically significant.

## 3 Result

### 3.1 Cytotoxicity of 20 Amino Acids Against HaCaT

The medium was formulated to include a suite of 15 amino acids, as detailed in Table S1. To test the potential effects of supplemental amino acids on cellular viability, we introduced additional amino acids into the culture medium at varying concentrations, ranging from 10 to 1000 μmol/L. The results showed that within the tested concentration range from 10 to 1000 μmol/L, the introduction of the 20 amino acids did not exhibit any significant toxic effects on HaCaT (Figure S1).

### 3.2 Effect of 20 Amino Acids on the ROS Level in HaCaT Induced by H_2_O_2_

**FIGURE 1:**
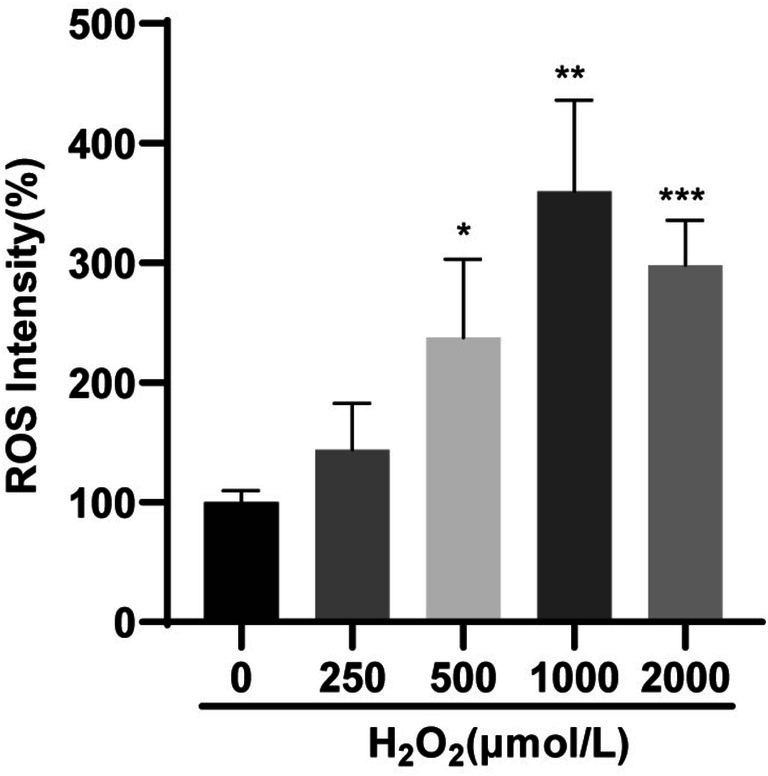
The establishment of oxidative stress model in HaCaT induced by H_2_O_2_. *p < 0.05, **p < 0.01, ***p < 0.001, compared to the 0 group without H_2_O_2_ treatment.

We initially established an oxidative stress model utilizing H_2_O_2_, culminating in the selection of a 1 mmol/L H_2_O_2_ concentration for the model (Figure 1). Subsequently, to investigate the antioxidant potential of 20 amino acids, we introduced amino acids with different concentrations into the established oxidative stress model. The results showed that Ala, Arg, Asp, Cys, Trp, Gly, Pro, Ile, Lys, Phe, Ser, Thr, Val, Glu, and Asn exhibited notable antioxidant capabilities (Figure S2).

### 3.3 Effect of 20 Amino Acids on the ROS Level in HaCaT Induced by UVB Irradiation

**FIGURE 2:**
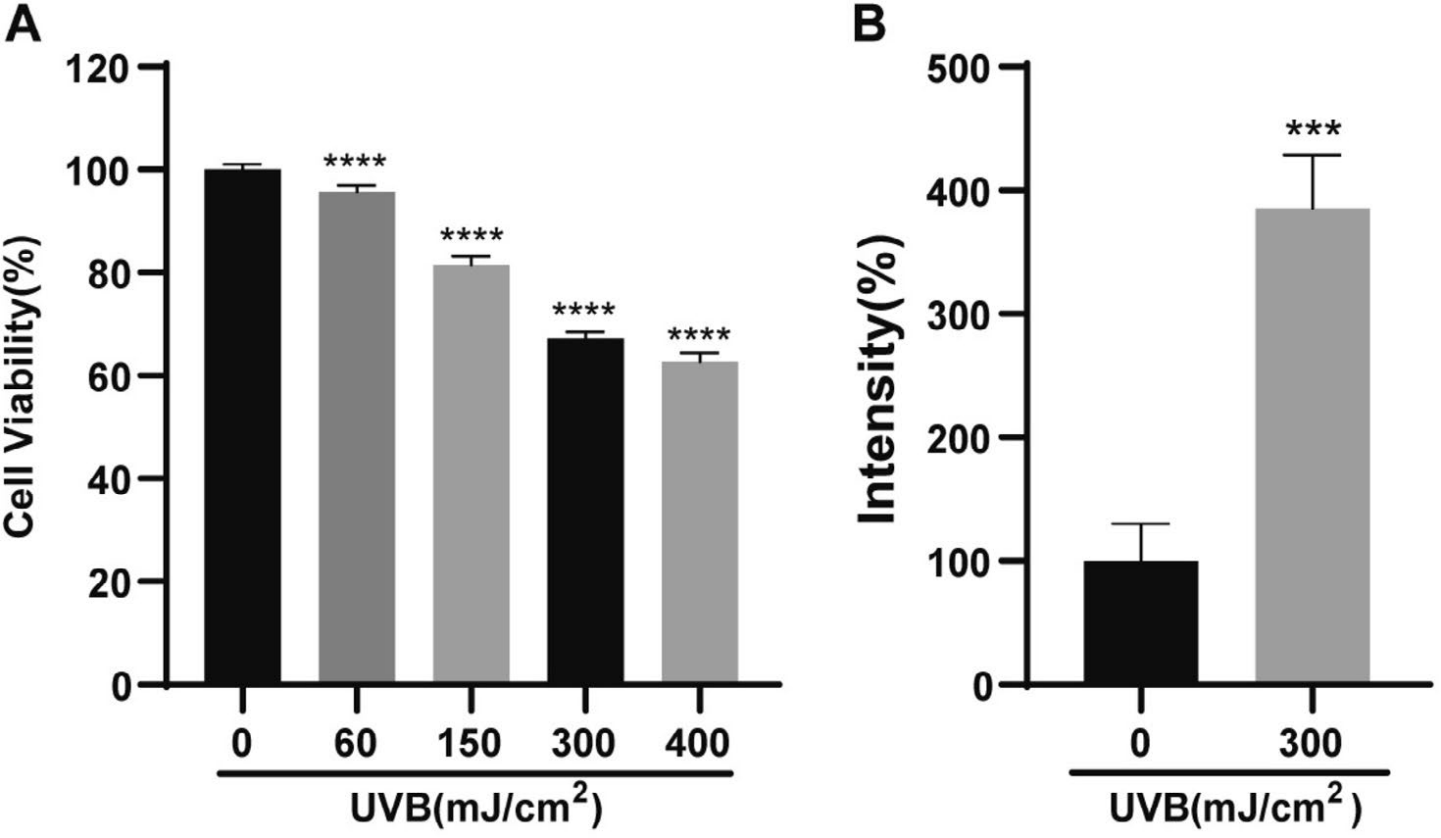
The establishment of photodamage model in HaCaT induced by UVB irradiation. (A) Effect of different doses of UVB irradiation on the viability ability of HaCaT. (B) Effect of indicated dose of UVB irradiation on ROS levels in HaCaT. Compared to the 0 group without UVB treatment, ***p < 0.001, ****p < 0.0001.

We detected the cell viability of HaCaT treated with different doses of UVB irradiation. The results showed that the survival rate of HaCaT decreased to about 60% at the dose of 300 mJ/cm^2^ (Figure 2A) and the ROS level of HaCaT induced by this dose of UVB increased significantly (Figure 2B). Therefore, the UVB model was established successfully and used for the subsequent evaluation.

Under the condition of UVB irradiation, the amino acid including Ala, Arg, Asp, Gln, Cys, Trp, Pro, His, Ile, Lys, Phe, Ser, Met, Thr, Val, Leu and Asn, had the ability to decrease the ROS level in HaCaT (Figure S3), which was increased by UVB irradiation, indicating that these amino acids showed potential protection against oxidative stress in photodamage.

### 3.4 Ten Potential Bioactive Tripeptides Selection Criteria Based on Molecular Docking Results

Tripeptides were screened based on the following criteria for molecular docking results: (1) the high binding energy; (2) peptides containing antioxidant amino acid residue based on biochemical and cellular level assays (e.g., Trp); (3) water solubility (e.g., octanol/water partition coefficient, ClogP); (4) structural analogs (e.g., similar results between Phe and Tyr). Additionally, considering the poor water solubility of Tyr, it was substituted with Phe. Ten tripeptides were selected, with their binding energies presented in Table S3 and their 2D binding diagrams depicted (Figures S4–S7). Subsequently, we validated the enzyme inhibitory activity of the ten tripeptides on the tyrosinase, hyaluronidase, and elastase. The results showed certain enzyme inhibitory activity of the ten tripeptides on the tyrosinase, hyaluronidase and elastase (Figure S8). We also investigated the effects of the ten tripeptides on HA, elastin and melanin content change. The results indicated that, compared with UVA group without tripeptides treatment, all ten tripeptides increased HA content while the increasement was moderate and the trend was consistent (Figure S9A). Simultaneously, under UVA irradiation, treatment with tripeptide HWW, WWH, HFW, FAW, WWT and RWW resulted in increased elastin content compared to the untreated UVA‐irradiated group, albeit with a modest magnitude of elevation (Figure S9B). In addition, tripeptide WWH, FRW, TWW, WRW, RWW, and RWF possess a certain ability to reduce melanin content in B16‐F10 cells (Figure S9C).

### 3.5 Migration Ability and Antioxidant Capacity of HaCaT of Ten Tripeptides

**FIGURE 3:**
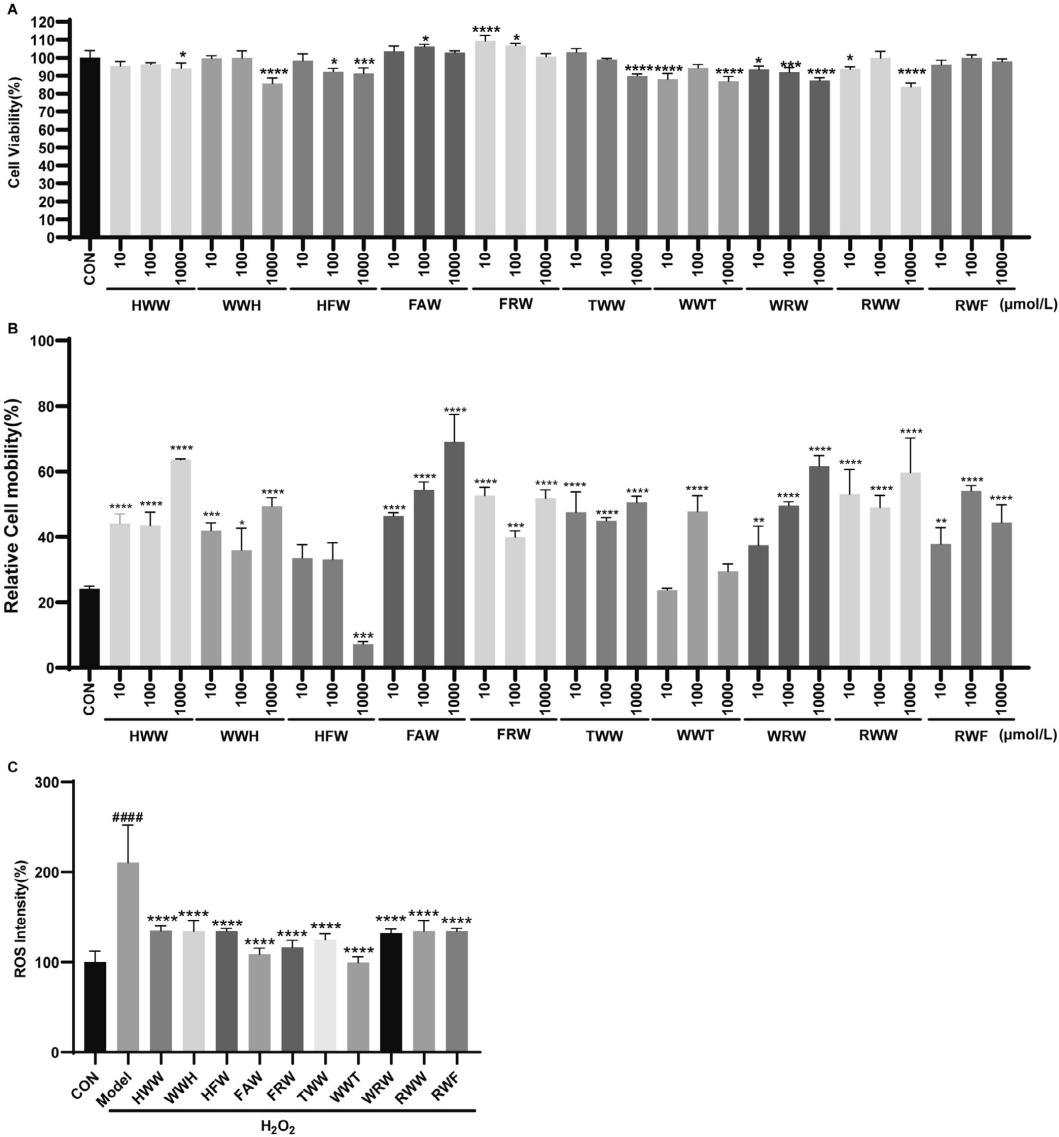
Effects of ten tripeptides on migration ability and antioxidant capacity of HaCaT. (A) Cell viability; (B) Migration ability. Compared to the CON group, *p < 0.05, **p < 0.01, ***p < 0.001, ****p < 0.0001. (C) Antioxidant capacity. Compared to the CON group, ^####^ *p <* 0.0001, Compared to the Model group, ****p < 0.0001.

Another focus of our study was the migration ability and antioxidant capacity of 10 tripeptides, which was preceded by safety testing. 90% cell viability was set as the safety screening criterion; we found that 1000 μmol/L of tripeptide WWH, TWW, WWT, WRW, and RWW exhibited certain toxicity to HaCaT (Figure 3A). Meanwhile, we investigated the effects of the ten tripeptides on the migration ability of HaCaT. The results showed that HWW, FAW, and WRW significantly promoted cell migration in a dose‐dependent manner, among which FAW showed the strongest migratory promotion. By contrast, HFW and WWT at a high concentration of 1000 μmol/L inhibited or had no effect on HaCaT cell migration (Figure 3B and Figure S10). Additionally, we selected 100 μmol/L as the concentration for detecting cellular antioxidant capacity. The results demonstrated that all ten tripeptides possessed antioxidant activity, and FAW and WWT reduced the ROS level in HaCaT to that of normal cells (Figure 3C). Based on the enzyme inhibitory activity, the content change of HA, elastin, and melanin, as well as cytotoxicity, cell migration, and antioxidant activity mentioned above, we ultimately chose tripeptide FAW for further investigation.

### 3.6 FAW Ameliorated the Photoaging in UVB‐Induced HaCaT

**FIGURE 4:**
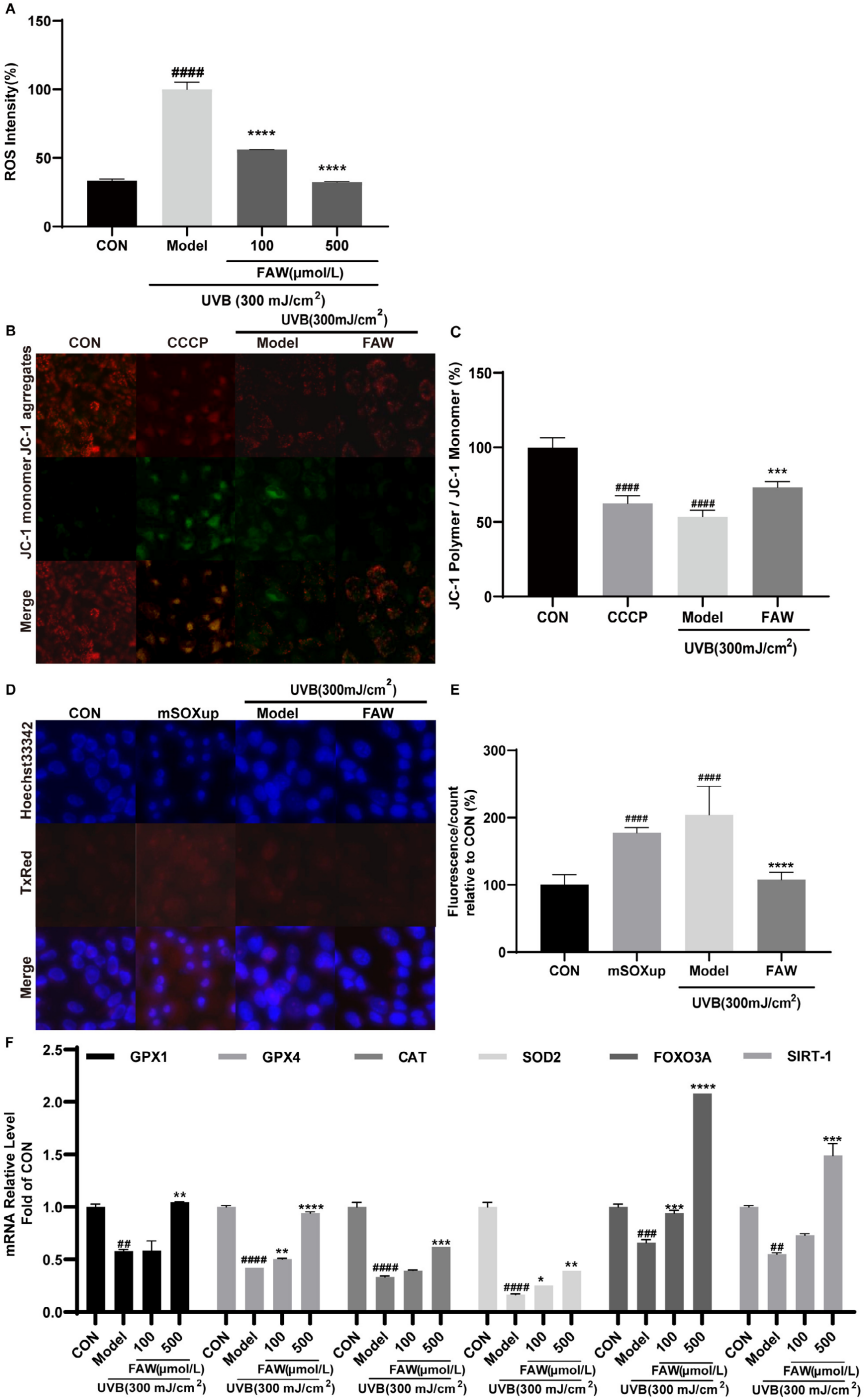
FAW reduced ROS intensity, repaired mitochondria and lowered the level of mitochondrial superoxide anions, and upregulated the expression of antioxidant‐related genes. (A) FAW reduced ROS intensity induced by UVB. (B) Visualization results of the effects of FAW on mitochondrial membrane potential in UVB‐induced HaCaT, and (C) the corresponding quantification results. (D) Visualization results of the effects of FAW on mitochondrial superoxide potential in HaCaT induced by UVB, and (E) the corresponding quantification results. (F) RT‐qPCR analysis of the expression of GPX1, GPX4, CAT, SOD2, FOXO3A and SIRT1 in HaCaT. Compared with CON group, ^##^ *p* < 0.01, ^###^ *p* < 0.001, ^####^ *p* < 0.0001. Compared with Model group, *p < 0.05, **p < 0.01, ***p < 0.001, ****p < 0.0001.

Given that UV radiation induces excessive production of ROS and causes mitochondrial damage in skin cells, we further evaluated the effects of FAW on intracellular ROS levels, mitochondrial membrane potential, and mitochondrial superoxide anion levels under UVB radiation exposure. UVB irradiation significantly increased ROS production in HaCaT, induced mitochondrial impairment, decreased mitochondrial membrane potential, and elevated mitochondrial superoxide anion levels. In contrast, FAW treatment reduced UVB‐induced elevation of ROS levels (Figure 4A), alleviated mitochondrial damage (Figure 4B,C), and reduced mitochondrial superoxide anion accumulation in HaCaT (Figure 4D,E). Additionally, we assessed the mRNA expression profiles of antioxidant‐related genes and observed that UVB irradiation down‐regulated the transcriptional levels of GPX1, GPX4, CAT, SOD2, FOXO3A and SIRT1 (Figure 4F), while FAW treatment upregulated their mRNA expression in a concentration‐dependent manner. These results collectively indicate that peptide FAW exerts a protective effect against UVB‐induced photodamage in skin cells.

### 3.7 Evaluation of Anti‐Aging Capacity of FAW in HaCaT

**FIGURE 5:**
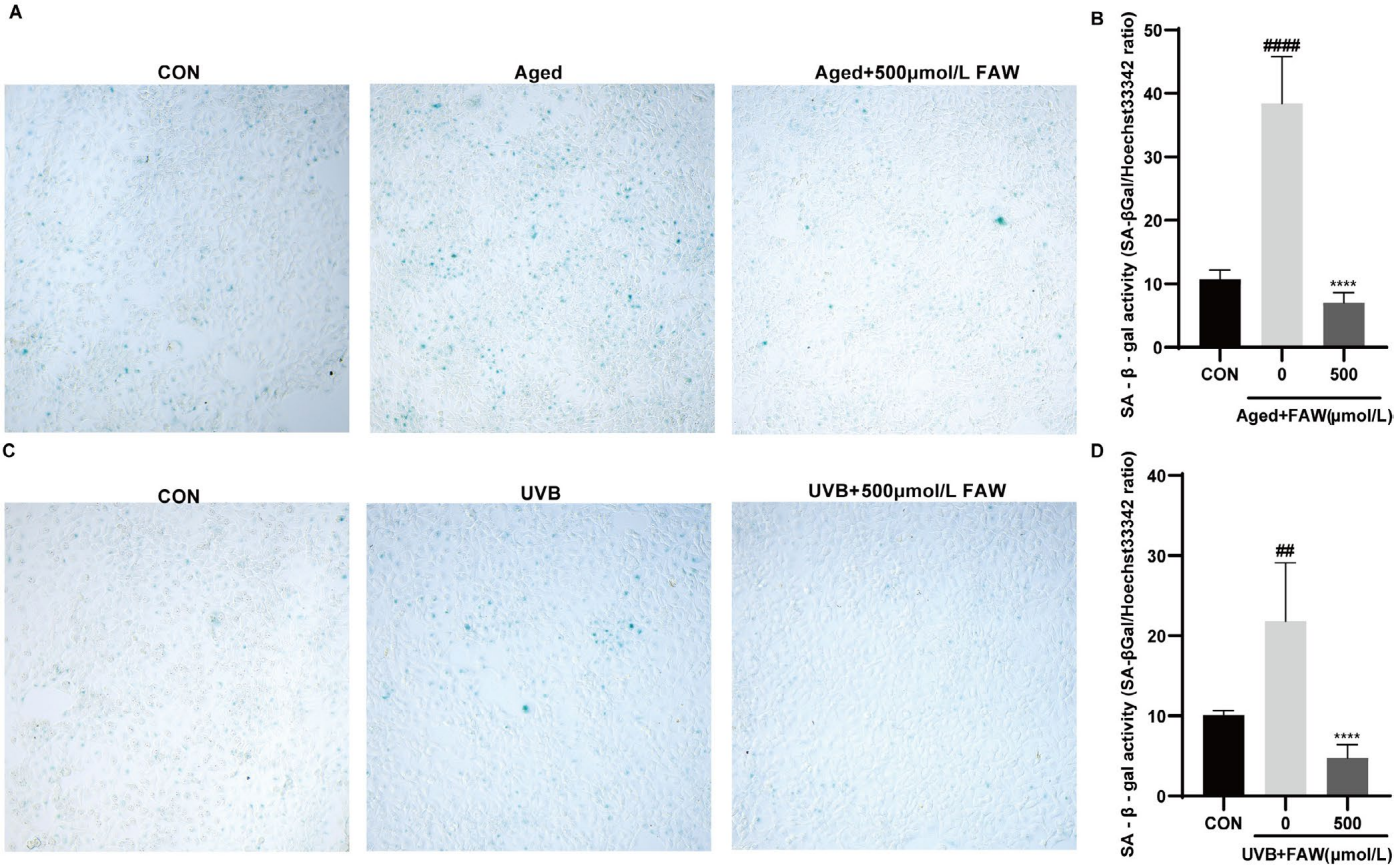
FAW reduced SA‐β‐gal staining‐positive rate of aged‐HaCaT. (A) Visualization results of the effect of FAW on the positive staining level of SA‐β‐Gal in passage number‐induced replicative senescence cells, and (B) the corresponding quantification results. (C) Visualization results of the effect of FAW on the positive staining level of SA‐β‐Gal in UVB‐induced photoaging cells, and the (D) corresponding quantification results. Compared with CON group, ^####^ *p <* 0.0001, Compared with 0 group, ****p < 0.0001.

To investigate the anti‐aging effects of FAW, we established two cellular senescence models: a passage number‐induced replicative senescence and a UVB‐induced photoaging model. SA‐β‐Gal staining revealed a significant increase in senescence‐positive cells in both models. Notably, FAW treatment markedly reduced the SA‐β‐gal staining‐positive rate of senescent cells, demonstrating its anti‐senescence capacity. These two models approach combining intrinsic aging and extrinsic stress‐induced senescence comprehensively validated the anti‐aging potential of FAW at the cellular level (Figure 5A–D).

### 3.8 RNA‐Seq Results:GO Enrichment and KEGG Pathway Analysis Verification

**FIGURE 6:**
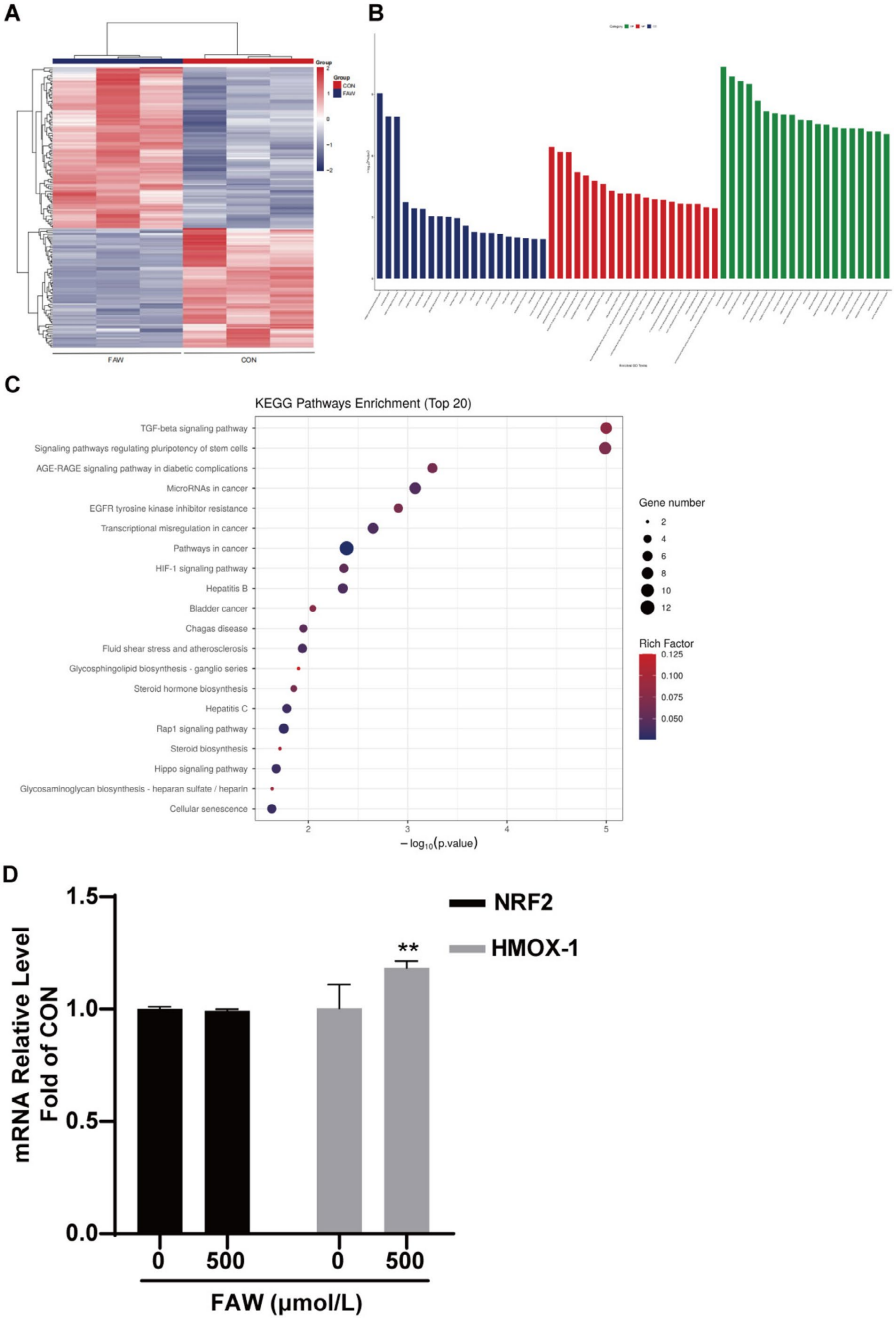
Quality assessment of reads and DEGs identified. (A) Heatmap of DEGs; (B) GO DEGs between FAW and CON; (C) KEGG of DEGs between FAW and CON; (D) RT‐qPCR analysis of the expression of NRF2 and HMOX‐1 in HaCaT. Compared with 0 group, **p < 0.01.

To better understand the effects of FAW (500 μmol/L), mRNA‐sequencing (*n* = 3) was completed by applied protein technology. A total of 164 differentially expressed genes (DEGs) were identified between the FAW group and the control group, with 70 down‐regulated and 94 upregulated (Figure 6A). Among the GO (biological process) enrichment (FAW vs. CON, *p* < 0.05), the tissue development, developmental process, cellular developmental process, cell differentiation, cellular response to organic substance, positive regulation of response to stimulus, regulation of cell population proliferation, animal organ development, response to organic substance, and positive regulation of developmental process were dominant. The top 30 enriched GO terms were listed (Figure 6B), concluding the top 10 biological processes, the top 10 cellular components, and molecular function, respectively.

KEGG were identified in which DEGs were significantly enriched. Among the pathways (FAW vs. CON, *p* < 0.05). TGF−β signaling pathway, signaling pathways regulating pluripotency of stem cells, AGE−RAGE signaling pathway in diabetic complications, MicroRNAs in cancer, EGFR tyrosine kinase inhibitor resistance, transcriptional misregulation in cancer, pathways in cancer and HIF−1 signaling pathway were dominant. The top 20 enriched KEGG terms were listed in Figure 6C. According to the results of KEGG, we found that the TGF‐β signaling pathway is indeed closely related to NRF2. TGF‐β induces NRF2 activation through ROS production. Notably, the downstream antioxidant gene HMOX‐1 of NRF2 was upregulated in DEGs, and the upregulated expression of HMOX‐1 gene was confirmed through RT‐qPCR (Figure 6D).

### 3.9 Evaluation of FAW in HSF

**FIGURE 7:**
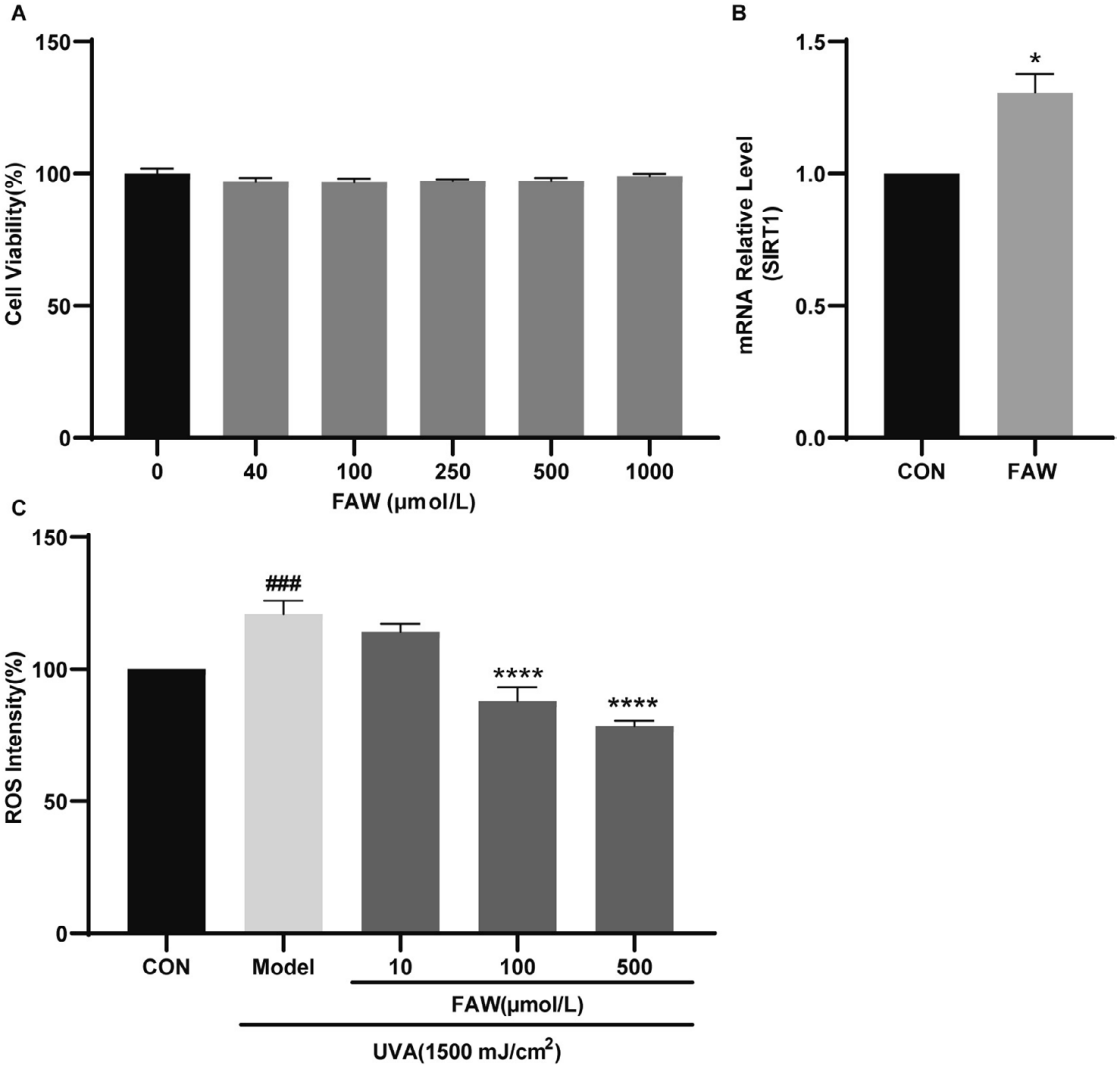
The effects of FAW on HSF cells. (A) Cell viability of HSF treated with FAW at different concentrations. (B) RT‐qPCR analysis of the expression of SIRT1 in HSF. (C) ROS intensity in HSF under UVA irradiation. *p < 0.05, **p < 0.01, ***p < 0.001, ****p < 0.0001.

We also conducted studies on FAW using HSF. The results showed that FAW exhibited no significant toxicity to HSF (Figure 7A). Subsequently, we detected the mRNA levels of anti‐aging‐related genes and found that FAW treatment upregulated the mRNA levels of SIRT1 in HSF (Figure 7B). Following FAW treatment, the ROS levels in HSF elevated by UVA irradiation were reduced (Figure 7C).

## 4 Discussion

Firstly, in this study, 10 potential bioactive tripeptides were screened out based on the results of antioxidant activity screening of amino acids and molecular docking. Secondly, we further screened out FAW by evaluating the antioxidant activity, pro‐migratory activity, and other relevant biological activities of these tripeptides. Thirdly, this study systematically investigated the ameliorative effects of FAW on UVB‐induced skin damage and photoaging, preliminarily explored its underlying molecular mechanism, and further extended the research to examine the repair effect of FAW on UVA‐induced photodamage in HSF. This work provides an experimental basis for the screening and application of bioactive molecules targeting skin photodamage and photoaging.

This study initially screened amino acids capable of reducing ROS production in HaCaT induced by H_2_O_2_ and UVB under safe concentrations, aiming to find tripeptide with antioxidant and anti‐aging properties. Through molecular docking and functional assays in HaCaT, the pleiotropic active peptide FAW was identified. In the screening of antioxidant activity of amino acids, 17 amino acids were chosen. With efficacy‐based skincare as the starting point, target proteins were selected based on their key roles in skin physiology: Tyrosinase was chosen as the whitening target, as it is the rate‐limiting enzyme in cutaneous melanogenesis, directly regulating melanin production [22]. Elastase was selected as the skin barrier repair target. UV‐induced excessive expression of elastase degrades the elastin in the dermis, accelerating the appearance of wrinkles and sagging. Hyaluronidase was selected as the moisturizing target, considering that HA, a major glycosaminoglycan in the ECM, functions as a critical “filler” and “water‐retention core” in the ECM network, and its degradation is closely associated with elastase activity [23]. NRF2 and its endogenous inhibitor Keap1 act as intracellular defense mechanisms to counteract oxidative stress. They are isolated in the cytoplasm by Keap1 and targeted for proteasomal degradation under basal conditions. In the case of oxidative stress, NRF2 is separated from Keap1 and translocates to the nucleus, activating the downstream NRF pathway [24]. NRF2 was selected as the antioxidant target, given its role as a core transcription factor in cellular antioxidant defense, which regulates at least 6 direct antioxidant pathways to establish a multi‐layered defense network against oxidative stress through mechanisms such as activating antioxidant enzymes, promoting regeneration of antioxidant substances, and protecting mitochondrial function [25]. A library of 4913 tripeptides generated by random combination of the 17 amino acids was subjected to molecular docking against the four targets mentioned above.

Experimental validation revealed that although some tripeptides exhibited certain enzyme inhibitory activity, the inhibitory efficacy of these 10 tripeptides against tyrosinase, hyaluronidase, and elastase were relatively low. Additional assays also showed that these tripeptides had some certain effects on the content change of HA, elastin, and melanin. Notably, the 10 tripeptides were found to promote HaCaT migration and reduce H_2_O_2_‐induced ROS levels. Integrating the results of enzyme inhibitory activity assays, and the content determination of HA, elastin and melanin, as well as migration experiments, and antioxidant tests, FAW was selected for in‐depth investigation of its cellular antioxidant mechanism.

Since a large amount of ROS is generated under UV irradiation, we employed the UVB‐induced photodamage model to detect the ROS‐scavenging effect of FAW on cells under a certain dose of UVB irradiation. The results showed that FAW exhibited ROS‐scavenging activity at both 100 μmol/L and 500 μmol/L concentrations.

A large number of researches have indicated that ROS are by‐products of mitochondrial metabolism. Mitochondria are not only the main source of ROS, but also play a crucial role in cell signal transduction and apoptosis [26]. UV irradiation caused mitochondrial damage, which in turn leads to progressive cell dysfunction [27, 28]. For this reason, we conducted an in‐depth study to explore whether the antioxidant property of FAW is associated with mitochondria. After UVB irradiation, FAW was added for treatment, and mitochondrial membrane potential as well as mitochondrial ROS (mtROS) were detected. The results demonstrated that FAW could effectively restore the membrane potential damaged by UVB irradiation and scavenge mtROS, suggesting that FAW may exert its antioxidant effect by acting on related proteins in mitochondria.

Studies have shown that when cells undergo oxidative stress, mitochondria first scavenge ROS through SOD [29, 30]. Among them, SOD2 is mainly localized in the mitochondrial matrix. After SOD2 scavenges ROS, the generated H_2_O_2_ needs to be further metabolized by glutathione peroxidase and catalase, such as GPX1, GPX4 and CAT [30, 31]. Therefore, after UVB irradiation, FAW was added for treatment, we detected the mRNA levels of GPX1, GPX4 and CAT. The results revealed that FAW could increase the mRNA levels of GPX1, GPX4 and CAT at both 100 μmol/L and 500 μmol/L concentrations, verifying that FAW exerts its antioxidant effect by regulating related proteases to mitochondria.

Studies have been reported that NRF2 has an indirect interaction with SIRT1, and SIRT1 can deacetylate FOXO3A to promote the expression of SOD2 under oxidative stress [32, 33]. Therefore, we detected the mRNA levels of SIRT1 and FOXO3A in HaCaT treated with FAW under UVB irradiation conditions. Since SIRT1 is also known as the “longevity gene,” we also detected the activity of SA‐β‐gal [34]. The results showed that FAW could increase the mRNA levels of SIRT1 and FOXO3A at both 100 μmol/L and 500 μmol/L, and reduce the SA‐β‐gal activity in senescent cells.

Combining the results mentioned above, we hypothesized that FAW may promote the expression of SOD2 under oxidative stress by regulating SIRT1 to deacetylate FOXO3A, thereby increasing the expression levels of GPX1, GPX4, and CAT to exert its antioxidant effect.

This study further investigated how FAW regulated the relevant mechanism of action through transcriptomics. The results showed that after FAW treatment, 164 DEGs were identified, among which multiple genes were related to the antioxidant pathway, such as NRF2 and HMOX‐1 [35, 36]. RT‐qPCR analysis confirmed that the result of HMOX‐1 was consistent with that of transcriptomics, while there was no significant change in NRF2. The main reason may be that FAW treatment has no effect on the mRNA level of NRF2. Instead, it exerted an antioxidant effect mainly by activating NRF2 and guiding it into the nucleus. Therefore, subsequent verification can be conducted by comparing the levels of NRF2 nuclear protein and cytoplasmic protein.

The aforementioned results demonstrate that the tripeptide FAW exhibits significant antioxidant activity in HaCaT cells. However, whether FAW also has an antioxidant effect on dermal fibroblasts deserves further in‐depth investigation. The results showed that FAW can significantly upregulate the mRNA levels of SIRT1 (“longevity gene”). To explore whether FAW can effectively ameliorate UVA‐induced photodamage in HSF, we established a UVA‐induced photodamage model of HSF. After UVA irradiation, FAW was added for treatment. The results showed that FAW could markedly scavenge ROS in HSF induced by UVA, indicating that FAW can effectively repair photodamage. However, whether the antioxidant mechanism of FAW in dermal fibroblasts is the same as that in epidermal keratinocytes still requires further in‐depth research.

## 5 Conclusion

To develop bioactive peptide molecules for mitigating UV‐induced skin photodamage, this study established a screening strategy, including amino acid screening, tripeptide library construction, multi‐target molecular docking, and functional validation. From 20 common amino acids, 17 high active ones were screened via H_2_O_2_‐ and UVB‐induced oxidative stress models. A 4913‐molecule tripeptide library was built, and ten candidate tripeptides were screened by molecular docking with tyrosinase, elastase, hyaluronidase, and Keap1. Further, our study revealed that tripeptide FAW might activated the NRF2/HMOX‐1 pathway and had strong antioxidant activity. FAW could also alleviate UVB‐ or UVA‐induced skin cell damage. Moreover, FAW was able to ameliorate passage number‐induced replicative senescence or UV‐induced photoaging, and promoted cellular migration. This study provides a screening paradigm for developing skin photoaging intervention molecules and highlights FAW application potential for skincare.

## Author Contributions

Jianghua Lu: Conceptualization; data curation and analysis; investigation and writing. Jie Li and Liang Wang: review and editing manuscript. Liming Xia: Conceptualization; methodology; resources. Yang Wan and Qingyong Li: Conceptualization; methodology; investigation; data curation and analysis; writing manuscript.

## Funding

This study was funded by Guangzhou Yiyang Bio‐technology Co. Ltd. (Project No. YYXM‐2024‐02).

## Conflicts of Interest

The authors declare no conflicts of interest.

## Supporting information


**Table S1:** Amino acid concentration in DMEM.
**Table S2:** RT‐qPCR primer sequences.
**Table S3:** Binding energy of molecular docking with 10 tripeptides.
**Figure S1:** The cytotoxicity of 20 amino acids against HaCaT.
**Figure S2:** The effect of 20 amino acids on ROS level in HaCaT with 1 mmol/L H_2_O_2_ treatment.
**Figure S3:** The effect of 20 amino acids on ROS level in HaCaT after UVB irradiation.
**Figure S4:** The 2D dimensional visualization of molecular docking result based on the interaction between tyrosinase (2y9x) and 10 selected tripeptides.
**Figure S5:** The 2D dimensional visualization of molecular docking result based on the interaction between elastase (1bru) and 10 selected tripeptides.
**Figure S6:** The 2D dimensional visualization of molecular docking result based on the interaction between hyaluronidase (2pe4) and 10 selected tripeptides.
**Figure S7:** The 2D dimensional visualization of molecular docking result based on the interaction between Keap1 (2flu1) and 10 selected tripeptides.
**Figure S8:** Enzyme inhibitor activity detection of tripeptides.
**Figure S9:** The effect of designed tripeptides on the content changes of extracellular matrix components and melanin.
**Figure S10:** Visualization results of HaCaT cell migration ability affected by ten tripeptides.

## Data Availability

Research data are not shared.

## References

[1] H.‐Y. Jang , G.‐B. Kim , J.‐M. Kim , et al., “Fisetin Inhibits UVA‐Induced Expression of MMP‐1 and MMP‐3 Through the NOX/ROS/MAPK Pathway in Human Dermal Fibroblasts and Human Epidermal Keratinocytes,” International Journal of Molecular Sciences 24, no. 24 (2023): 17358. 38139186 10.3390/ijms242417358 PMC10743569

[2] J. D'Orazio , S. Jarrett , A. Amaro‐Ortiz , and T. Scott , “UV Radiation and the Skin,” International Journal of Molecular Sciences 14, no. 6 (2013): 12222–12248. 23749111 10.3390/ijms140612222 PMC3709783

[3] B. A. Gilchrest , “Photoaging,” Journal of Investigative Dermatology 133 (2013): E2–E6. 10.1038/skinbio.2013.176 23820721

[4] X. Tang , T. Yang , D. Yu , H. Xiong , and S. Zhang , “Current Insights and Future Perspectives of Ultraviolet Radiation (UV) Exposure: Friends and Foes to the Skin and Beyond the Skin,” Environment International 185 (2024): 185. 10.1016/j.envint.2024.108535 38428192

[5] N. E. Saadé , I. W. Nasr , C. A. Massaad , B. Safieh‐Garabedian , S. J. Jabbur , and S. A. Kanaan , “Modulation of Ultraviolet‐Induced Hyperalgesia and Cytokine Upregulation by Interleukins 10 and 13,” British Journal of Pharmacology 131, no. 7 (2009): 1317–1324. 10.1038/sj.bjp.0703699 PMC1572458 11090103

[6] O. Gag , Ș. Dinu , H. Manea , et al., “UVA/UVB Irradiation Exerts a Distinct Phototoxic Effect on Human Keratinocytes Compared to Human Malignant Melanoma Cells,” Life 13, no. 5 (2023): 1144. 37240789 10.3390/life13051144 PMC10221048

[7] S. E. Mancebo and S. Q. Wang , “Recognizing the Impact of Ambient Air Pollution on Skin Health,” Journal of the European Academy of Dermatology and Venereology 29, no. 12 (2015): 2326–2332. 26289769 10.1111/jdv.13250 PMC5916788

[8] A. Vierkötter , T. Schikowski , U. Ranft , et al., “Airborne Particle Exposure and Extrinsic Skin Aging,” Journal of Investigative Dermatology 130, no. 12 (2010): 2719–2726. 20664556 10.1038/jid.2010.204

[9] N. Xu , G. Chen , and H. Liu , “Antioxidative Categorization of Twenty Amino Acids Based on Experimental Evaluation,” Molecules 22, no. 12 (2017): 2066. 29186888 10.3390/molecules22122066 PMC6149856

[10] T. Z. Y. W. Shucheng He and J. L. D. Z. Yinshi Zhu , “Enhancing the In Vitro Antioxidant Capacities via the Interaction of Amino Acids,” Emirates Journal of Food and Agriculture 20 (2018): 224.

[11] J. I. Vandenberg , “The Assimilation of Tri‐ and Tetrapeptides by Human Erythrocytes,” Biochimica et Biophysica Acta 1985, no. 846 (1985): 127–134. 10.1016/0167-4889(85)90118-1 4016152

[12] T. Sato , T. Matsuda , K. Tagawa , and S. Segawa , “α‐Ketoglutarate Produced by Lactic Acid Bacteria Inhibits Hyaluronidase Activity,” Bioscience of Microbiota, Food and Health 43, no. 4 (2024): 391–400. 39364123 10.12938/bmfh.2024-017 PMC11444863

[13] M. E. Z. I. Orqueda , K. Bravo , E. Osorio , and M. I. Isla , “Potential Use of Native Fruits Waste From Argentina as Nonconventional Sources of Cosmetic Ingredients,” Journal of Cosmetic Dermatology 21, no. 10 (2022): 5058–5065. 35373450 10.1111/jocd.14959

[14] J. M. Andrade , E. M. Domínguez‐Martín , M. Nicolai , C. Faustino , L. M. Rodrigues , and P. Rijo , “Screening the Dermatological Potential of Plectranthus Species Components: Antioxidant and Inhibitory Capacities Over Elastase, Collagenase and Tyrosinase,” Journal of Enzyme Inhibition and Medicinal Chemistry 36, no. 1 (2020): 258–270. 10.1080/14756366.2020.1862099 PMC7808741 33322969

[15] A. Martín‐Martínez , N. Sánchez‐Marzo , D. Martínez‐Casanova , et al., “High Global Antioxidant Protection and Stimulation of the Collagen Synthesis of New Anti‐Aging Product Containing an Optimized Active Mix,” Journal of Cosmetic Dermatology 21, no. 9 (2022): 3993–4000. 35050544 10.1111/jocd.14703 PMC9788327

[16] L. Baird and M. Yamamoto , “The Molecular Mechanisms Regulating the KEAP1‐NRF2 Pathway,” Molecular and Cellular Biology 40, no. 13 (2023): 1254. 10.1128/MCB.00099-20 PMC7296212 32284348

[17] M. T. Y. Lam , W. Li , M. G. Rosenfeld , and C. K. Glass , “Enhancer RNAs and Regulated Transcriptional Programs,” Trends in Biochemical Sciences 39, no. 4 (2014): 170–182. 24674738 10.1016/j.tibs.2014.02.007 PMC4266492

[18] M. Rojo de la Vega , E. Chapman , and D. D. Zhang , “NRF2 and the Hallmarks of Cancer,” Cancer Cell 34, no. 1 (2018): 21–43. 29731393 10.1016/j.ccell.2018.03.022 PMC6039250

[19] J. H. Choi , S. Lee , H. J. Han , and J. Kwon , “Antioxidation and Anti‐Inflammatory Effects of Gamma‐Irradiated Silk Sericin and Fibroin in H_2_O_2_‐Induced HaCaT Cell,” Korean Journal of Physiology & Pharmacology 27, no. 1 (2023): 105–112. 36575938 10.4196/kjpp.2023.27.1.105 PMC9806640

[20] J. Eberhardt , D. Santos‐Martins , A. F. Tillack , and S. Forli , “AutoDock Vina 1.2. 0: New Docking Methods, Expanded Force Field, and Python Bindings,” Journal of Chemical Information and Modeling 61, no. 8 (2021): 3891–3898. 34278794 10.1021/acs.jcim.1c00203 PMC10683950

[21] C. J. Igbokwe , Y. Feng , H. Louis , et al., “Novel Antioxidant Peptides Identified From Coix Seed by Molecular Docking, Quantum Chemical Calculations and Invitro Study in HepG2 Cells,” Food Chemistry 440 (2024): 138234. 38145582 10.1016/j.foodchem.2023.138234

[22] T. Pillaiyar , M. Manickam , and V. Namasivayam , “Skin Whitening Agents: Medicinal Chemistry Perspective of Tyrosinase Inhibitors,” Journal of Enzyme Inhibition and Medicinal Chemistry 32, no. 1 (2017): 403–425. 28097901 10.1080/14756366.2016.1256882 PMC6010116

[23] X. Yang , K. Miao , Z. Chen , et al., “Oligomeric Hyaluronic Acid‐Modified Liposomes Effectively Improved Skin Permeability and Anti‐Ageing Activity of Ellagic Acid,” Scientific Reports 15, no. 1 (2025): 27183. 40715158 10.1038/s41598-025-06948-0 PMC12297256

[24] X. Sun , Z. Ou , R. Chen , et al., “Activation of the p62‐Keap1‐NRF2 Pathway Protects Against Ferroptosis in Hepatocellular Carcinoma Cells,” Hepatology 63, no. 1 (2015): 173–184. 26403645 10.1002/hep.28251 PMC4688087

[25] M. Yamamoto , T. W. Kensler , and H. Motohashi , “The KEAP1‐NRF2 System: A Thiol‐Based Sensor‐Effector Apparatus for Maintaining Redox Homeostasis,” Physiological Reviews 98, no. 3 (2018): 1169–1203. 29717933 10.1152/physrev.00023.2017 PMC9762786

[26] N. Jessica , A. S. Peoples , N. Ghazal , T. T. Pham , and J. Q. Kwong , “Mitochondrial dysfunction and oxidative stress in heart disease,” Experimental & Molecular Medicine 51 (2019): 162. 31857574 10.1038/s12276-019-0355-7 PMC6923355

[27] M. Jan , M. L. Suski , M. Bonora , P. Pinton , J. Duszynski , and M. R. Wieckowski , “Relation Between Mitochondrial Membrane Potential and ROS Formation. Mitochondrial Bioenergetics: Methods and Protocols,” Methods in Molecular Biology 810 (2012): 183–205. 22057568 10.1007/978-1-61779-382-0_12

[28] L. Ge , W. Chen , and F. Wei , “Annexin A1 Protects Epidermal Stem Cells Against Ultraviolet‐B Irradiation‐Induced Mitochondrial Dysfunction,” Archives of Dermatological Research 316, no. 7 (2024): 385. 38874830 10.1007/s00403-024-02875-8

[29] D. Wang , L. Cao , X. Zhou , et al., “Mitigation of Honokiol on Fluoride‐Induced Mitochondrial Oxidative Stress, Mitochondrial Dysfunction, and Cognitive Deficits Through Activating AMPK/PGC‐1α/Sirt3,” Journal of Hazardous Materials 437 (2022): 129381. 35752048 10.1016/j.jhazmat.2022.129381

[30] D. Jerotic , M. Matic , S. Suvakov , et al., “Association of Nrf2, SOD2 and GPX1 Polymorphisms With Biomarkers of Oxidative Distress and Survival in End‐Stage Renal Disease Patients,” Toxins 11, no. 7 (2019): 431. 31340563 10.3390/toxins11070431 PMC6669734

[31] Q. Li , J. Liao , W. Chen , et al., “NAC Alleviative Ferroptosis in Diabetic Nephropathy via Maintaining Mitochondrial Redox Homeostasis Through Activating SIRT3‐SOD2/Gpx4 Pathway,” Free Radical Biology and Medicine 187 (2022): 158–170. 35660452 10.1016/j.freeradbiomed.2022.05.024

[32] F. Wang , M. Nguyen , F. X. F. Qin , and Q. Tong , “SIRT2 Deacetylates FOXO3a in Response to Oxidative Stress and Caloric Restriction,” Aging Cell 6, no. 4 (2007): 505–514. 17521387 10.1111/j.1474-9726.2007.00304.x

[33] D. Zhou , Y. Ran , R. Yu , G. Liu , D. Ran , and Z. Liu , “SIRT1 Regulates Osteoblast Senescence Through SOD2 Acetylation and Mitochondrial Dysfunction in the Progression of Osteoporosis Caused by Cadmium Exposure,” Chemico‐Biological Interactions 382 (2023): 110632. 37451666 10.1016/j.cbi.2023.110632

[34] Y. Wang , Y. Liang , and P. M. Vanhoutte , “SIRT1 and AMPK in Regulating Mammalian Senescence: A Critical Review and a Working Model,” FEBS Letters 585, no. 7 (2011): 986–994. 21130086 10.1016/j.febslet.2010.11.047

[35] J. L. Zhong , C. M. Raval , M. F. Nisar , et al., “Development of Refractoriness of HO‐1 Induction to a Second Treatment With UVA Radiation and the Involvement of Nrf2 in Human Skin Fibroblasts,” Photochemistry and Photobiology 90, no. 6 (2014): 1340–1348. 25213834 10.1111/php.12343

[36] R. Rana , R. Mukherjee , S. Mehan , Z. Khan , G. Das Gupta , and A. S. Narula , “Molecular Mechanisms of Neuroprotection: The Interplay of Klotho, SIRT‐1, Nrf2, and HO‐1 in Neurological Health,” Behavioural Brain Research 485 (2025): 485. 10.1016/j.bbr.2025.115545 40120944

