# Supplementary material for: Identification of A Novel Antioxidant Tripeptide Ameliorating Skin Photoaging
Source: J Cosmet Dermatol. 2026 Jun 10;25(6):e70977. doi: 10.1111/jocd.70977 (PMC13254024; doi:10.1111/jocd.70977)
Supplement: Supplementary file 1 — Table S1: Amino acid concentration in DMEM. Table S2: RT‐qPCR primer sequences. Table S3: Binding energy of molecular docking with 10 tripeptides. Figure S1: The cytotoxicity of 20 amino acids against HaCaT. Figure S2: The effect of 20 amino acids on ROS level in HaCaT with 1 mmol/L H2O2 treatment. Figure S3: The effect of 20 amino acids on ROS level in HaCaT after UVB irradiation. Figure S4: The 2D dimensional visualization of molecular docking result based on the interaction between tyrosinase (2y9x) and 10 selected tripeptides. Figure S5: The 2D dimensional visualization of molecular docking result based on the interaction between elastase (1bru) and 10 selected tripeptides. Figure S6: The 2D dimensional visualization of molecular docking result based on the interaction between hyaluronidase (2pe4) and 10 selected tripeptides. Figure S7: The 2D dimensional visualization of molecular docking result based on the interaction between Keap1 (2flu1) and 10 selected tripeptides. Figure S8: Enzyme inhibitor activity detection of tripeptides. Figure S9: The effect of designed tripeptides on the content changes of extracellular matrix components and melanin. Figure S10: Visualization results of HaCaT cell migration ability affected by ten tripeptides. [file JOCD-25-e70977-s001.docx]

**Supplemental Information**

**Identification of A Novel Antioxidant Tripeptide Ameliorating Skin Photoaging**

**Supplemental Table 1.** Amino acid concentration in DMEM

| Amino Acid | mg/mL |
| --- | --- |
| Glycine | 30 |
| L-Alanyl-glutamine | 862 |
| L-Arginine hydrochloride | 84 |
| L-Cystine | 48 |
| L-Histidine hydrochloride-H2O | 42 |
| L-Isoleucine | 105 |
| L-Leucine | 105 |
| L-Lysine hydrochloride | 146 |
| L-Methionine | 30 |
| L-Phenylalanine | 66 |
| L-Serine | 42 |
| L-Threonine | 95 |
| L-Tryptophan | 16 |
| L-Tyrosine disodium salt dihydrate | 104 |
| L-Valine | 94 |

**Supplemental Table 2.** RT-qPCR primer sequences

| Gene | Forward primer (5′-3′) | Reverse primer (3′-5′) |
| --- | --- | --- |
| GPX1 | CAGTCGGTGTATGCCTTCTCG | GAGGGACGCCACATTCTCG |
| GPX4 | GAGGCAAGACCGAAGTAAACTAC | CCGAACTGGTTACACGGGAA |
| CAT | TGGGATCTCGTTGGAAATAACAC | TCAGGACGTAGGCTCCAGAAG |
| SOD2 | GGAAGCCATCAAACGTGACTT | CCCGTTCCTTATTGAAACCAAGC |
| FOXO3A | TCACGCACCAATTCTAACGC | CACGGCTTGCTTACTGAAGG |
| SIRT1 | TAGCCTTGTCAGATAAGGAAGGA | ACAGCTTCACAGTCAACTTTGT |
| NRF2 | TCAGCGACGGAAAGAGTATGA | CCACTGGTTTCTGACTGGATGT |
| HMOX-1 | AAGACTGCGTTCCTGCTCAAC | AAAGCCCTACAGCAACTGTCG |
| GAPDH | GTGGACCTGACCTGCCGTCT | GGAGGAGTGGGTGTCGCTGT |


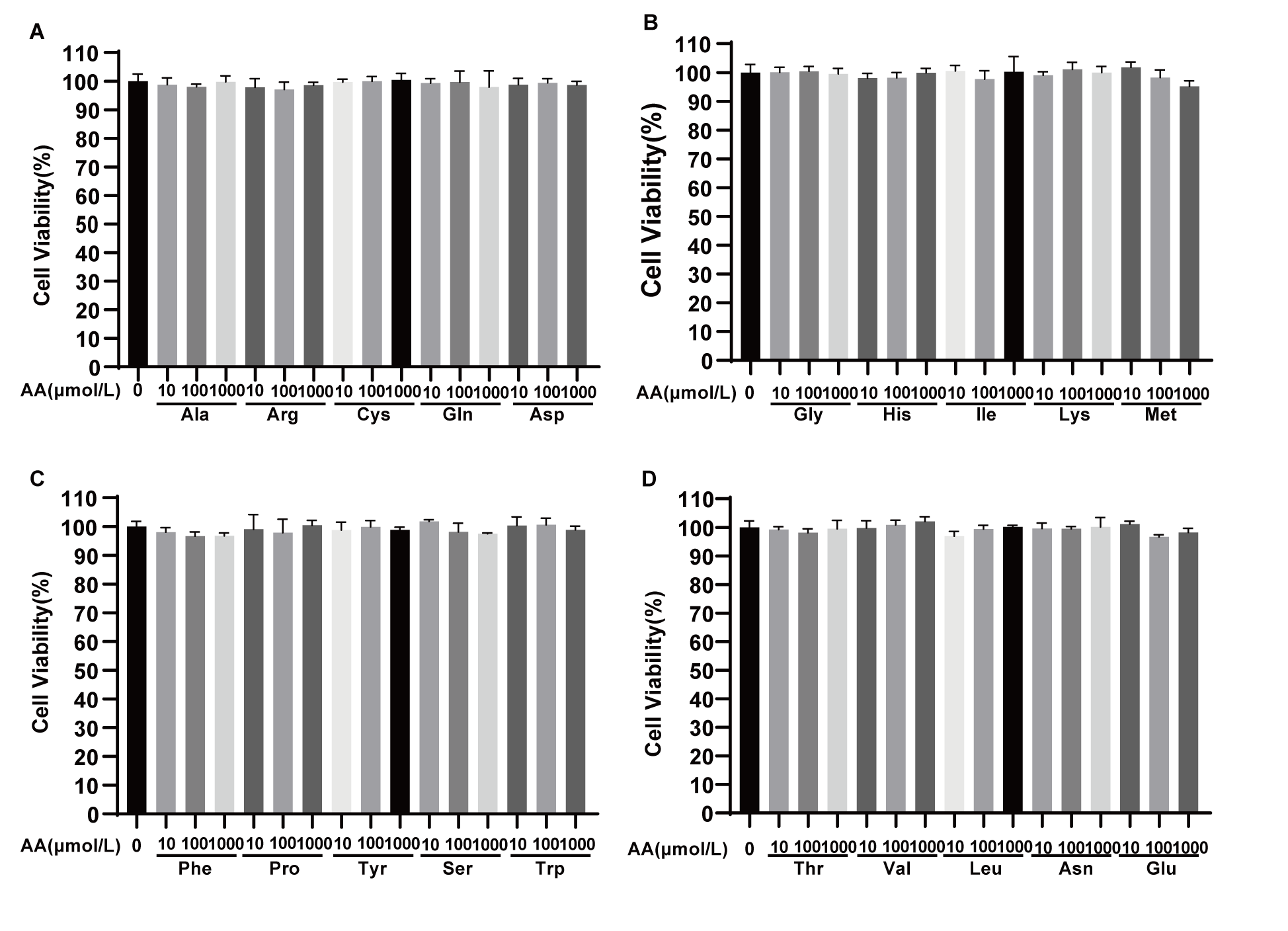
**Supplemental Figure 1.** The cytotoxicity of 20 amino acids against HaCaT. The cell viability of HaCaT treated with (A) Ala, Arg, Cys, Gln and Asp, (B) Gly, His, Ile, Lys and Met, (C) Phe, Pro, Tyr, Ser and Trp, (D) Thr, Val, Leu, Asn and Glu. AA: amino acid.


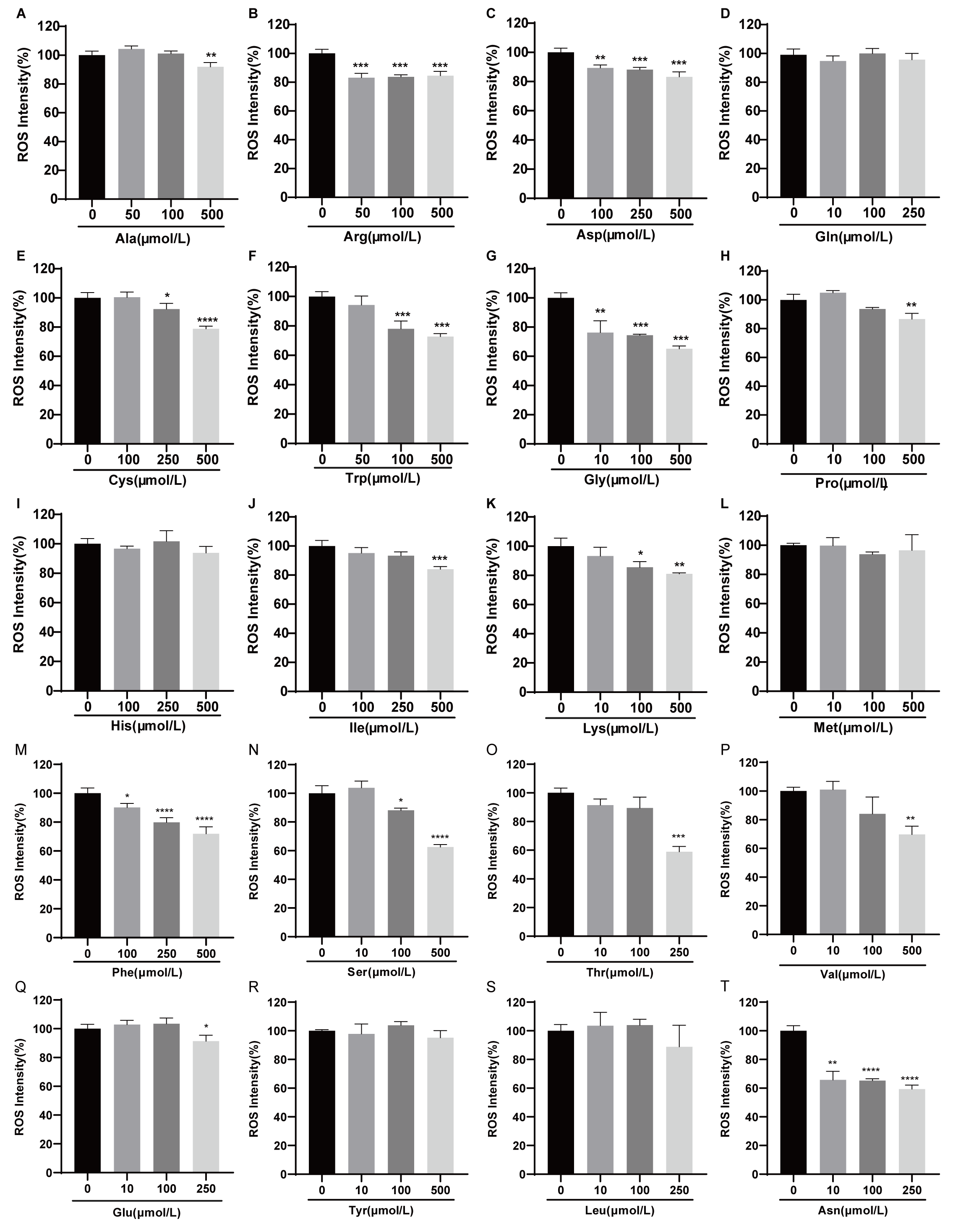


**Supplemental Figure 2.** The effect of 20 amino acids on ROS level in HaCaT with 1 mmol/L H_2_O_2_ treatment. Compared with 0 group, * *P* < 0.05, ** *P* < 0.01, *** *P* < 0.001, **** *P* < 0.0001.


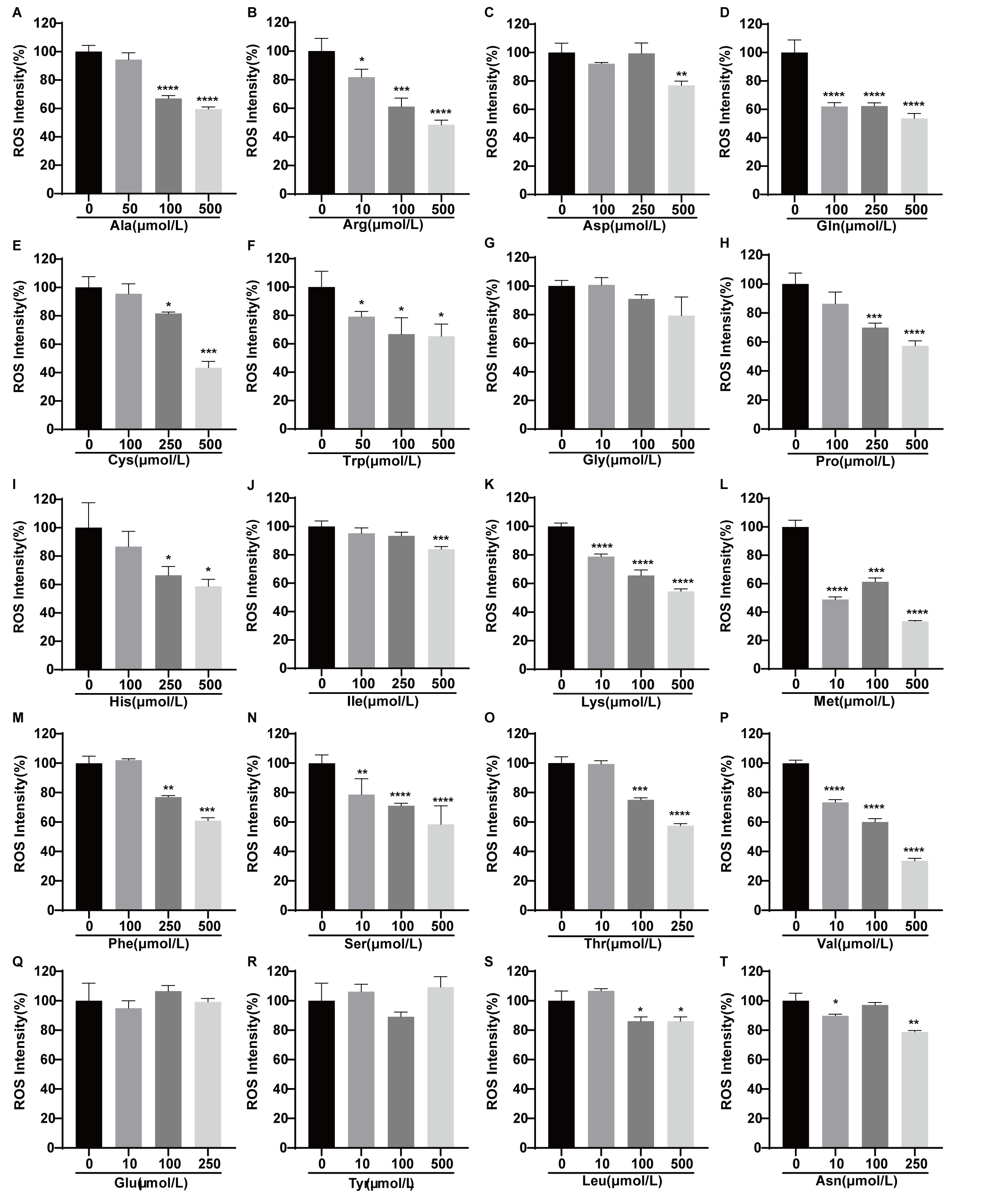


**Supplemental Figure 3.** The effect of 20 amino acids on ROS level in HaCaT after UVB irradiation. Compared with 0 group, * *P <* 0.05, ** *P <* 0.01, *** *P <* 0.001, **** *P <* 0.0001.

**Supplemental Table 3.** Binding energy of molecular docking with 10 tripeptides

| Ligand | Binding Energy (kcal/mol) | | | |
| --- | --- | --- | --- | --- |
|  | Tyrosinase (2y9x) | Elastase (1bru) | Hyaluronidase (2pe4) | Keap1 (2flu1) |
| HWW | -8.8 | -8.4 | -9.8 | -10.72 |
| WWH | -8.5 | -7.8 | -9.8 | -10.7 |
| HFW | -8.6 | -8.1 | -9.5 | -6.893 |
| FAW | -8.8 | -7.1 | -10 | -8.825 |
| FRW | -8.7 | -7.5 | -10.1 | -9.639 |
| TWW | -8.8 | -8.3 | -9.4 | -9.863 |
| WWT | -7.9 | -7.7 | -10.3 | -9.095 |
| WRW | -9.6 | -8.2 | -9.7 | -10.76 |
| RWW | -9.1 | -8.5 | -8.9 | -10.82 |
| RWF | -8.4 | -8.4 | -9.5 | -9.473 |


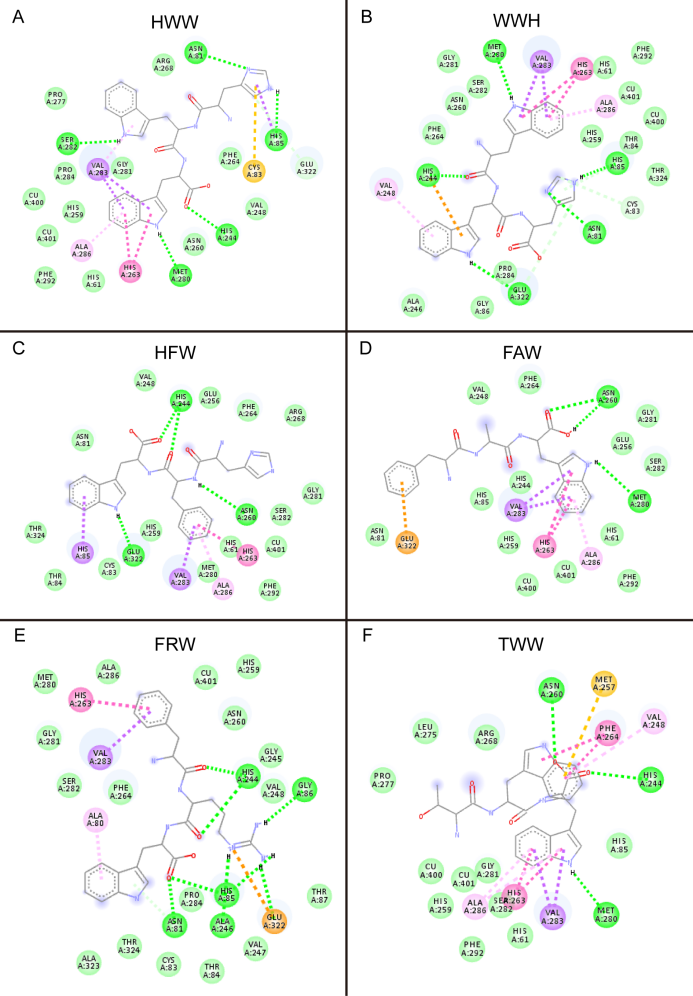

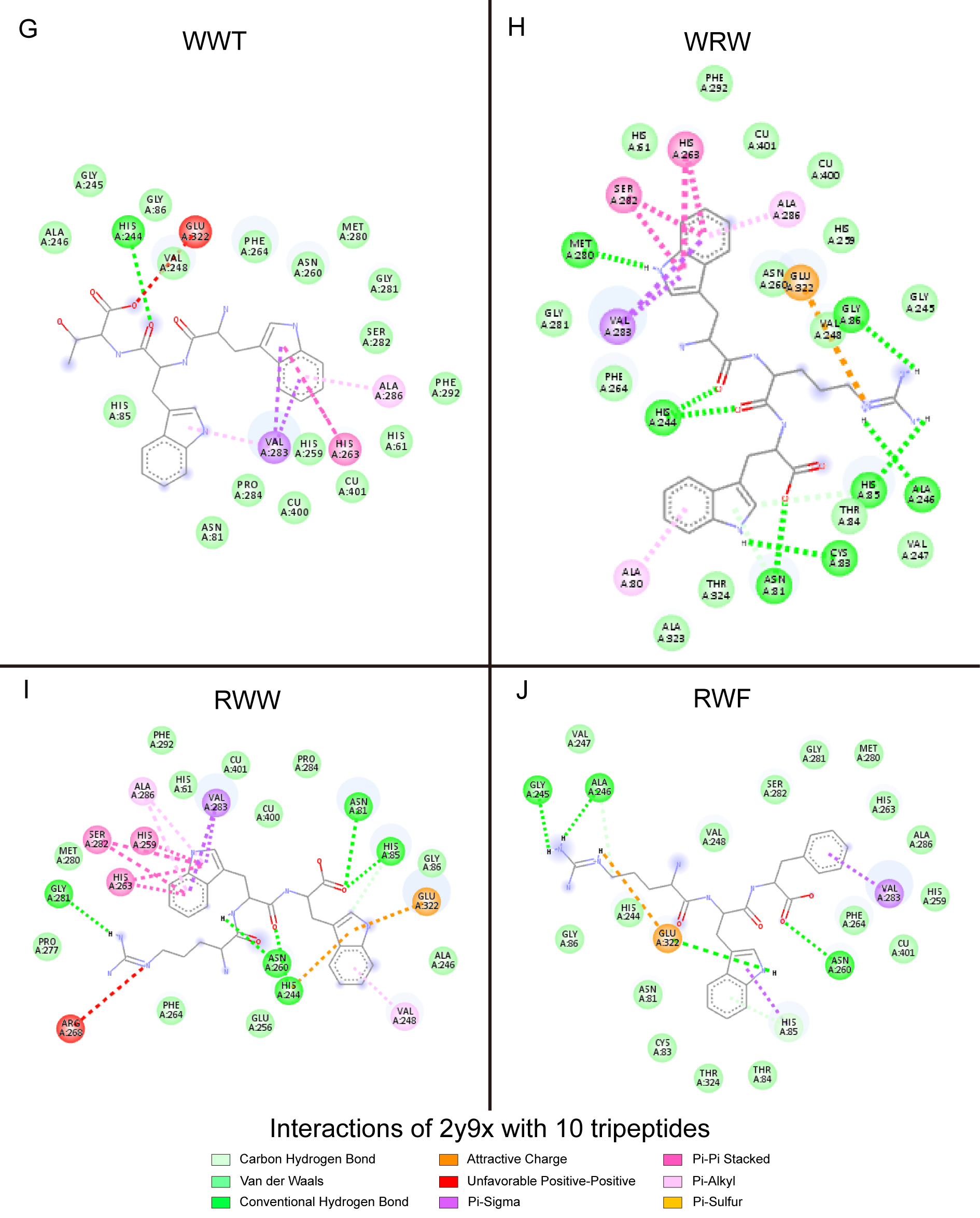


**Supplemental Figure 4.** The 2D dimensional visualization of molecular docking result based on the interaction between tyrosinase (2y9x) and 10 tripeptides (HWW, WWH, HFW, FAW, FRW, TWW, WWT, WRW, RWW, RWF).


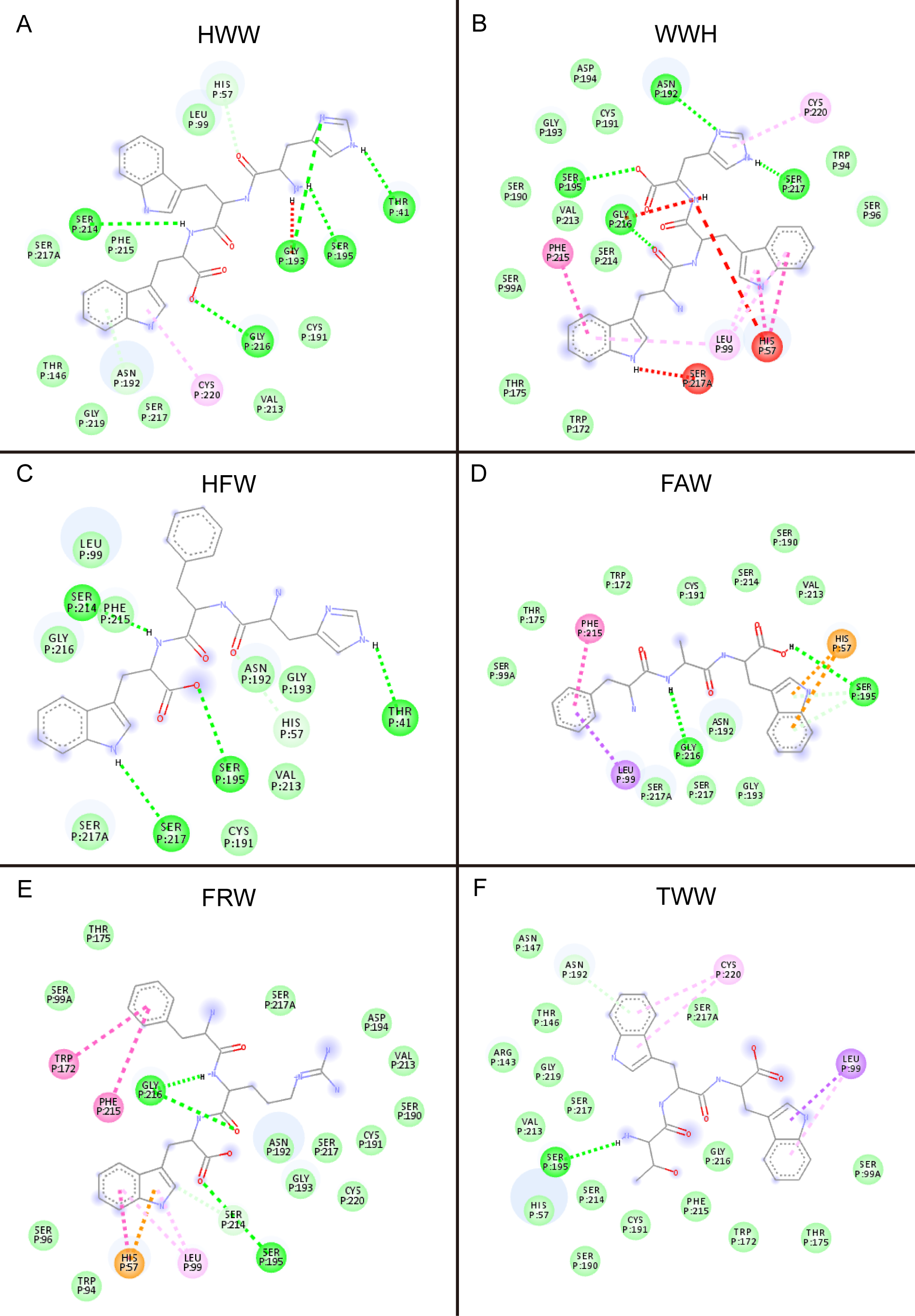

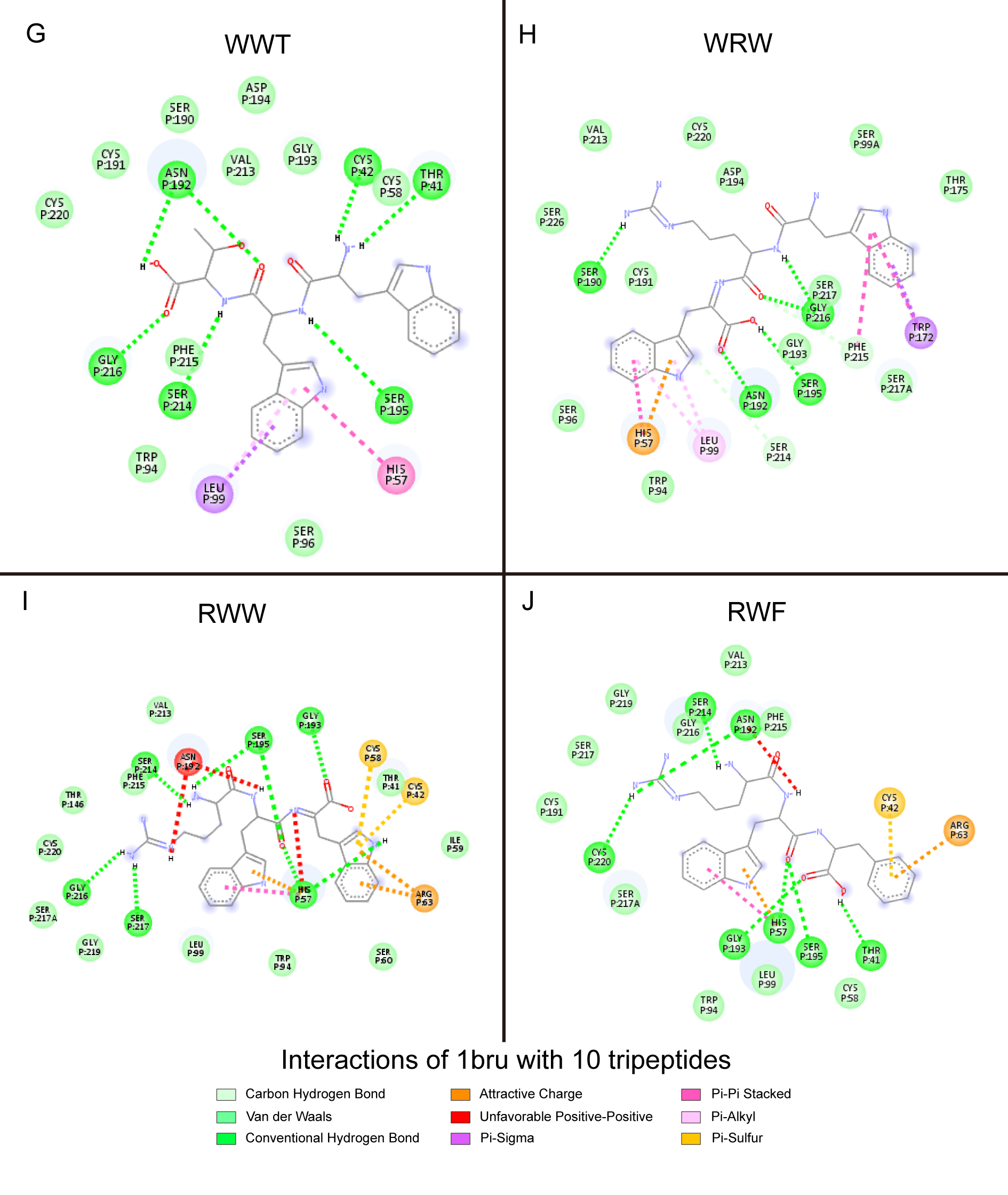


**Supplemental Figure 5.** The 2D dimensional visualization of molecular docking result based on the interaction between elastase (1bru) and 10 tripeptides (HWW, WWH, HFW, FAW, FRW, TWW, WWT, WRW, RWW, RWF).


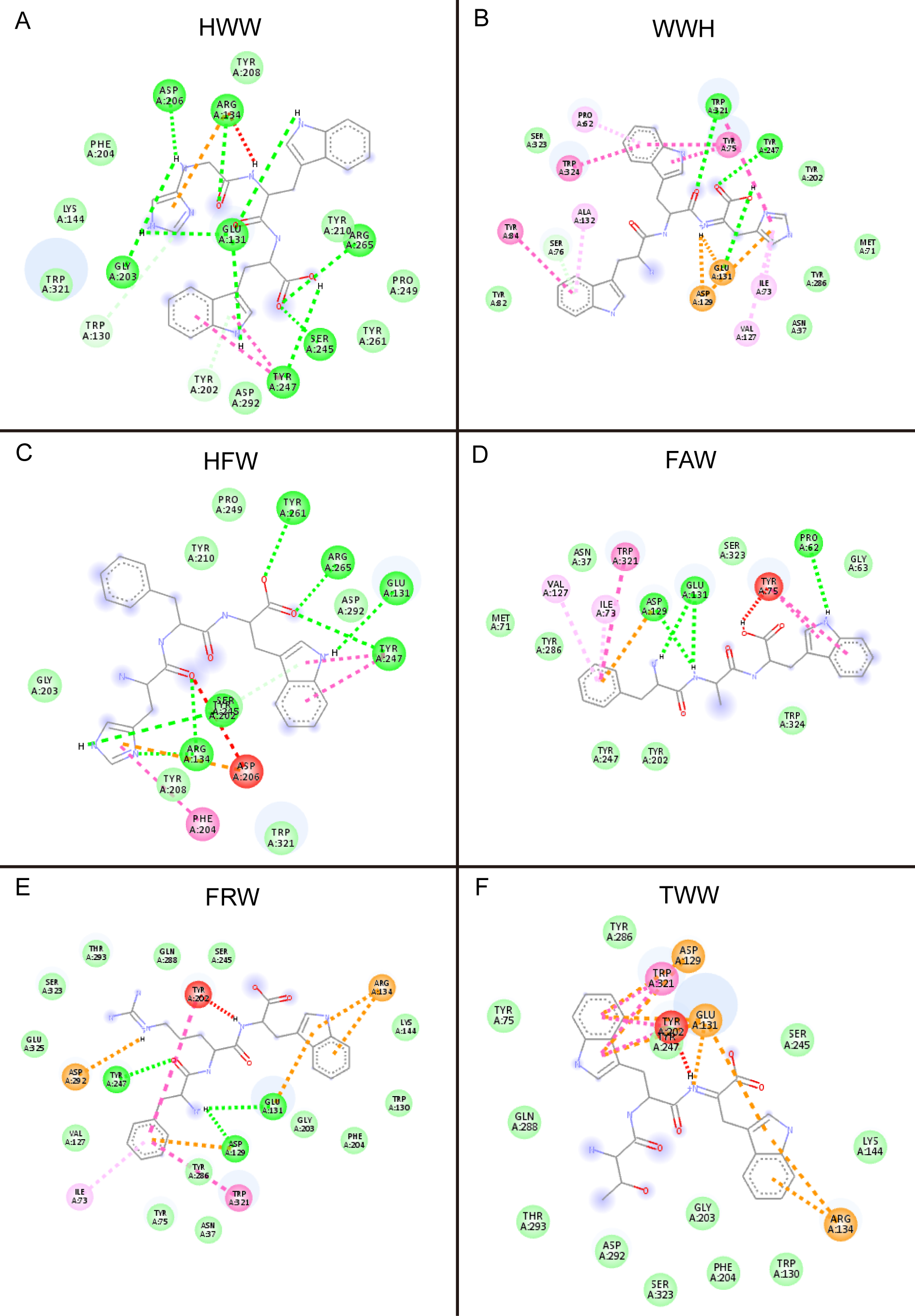

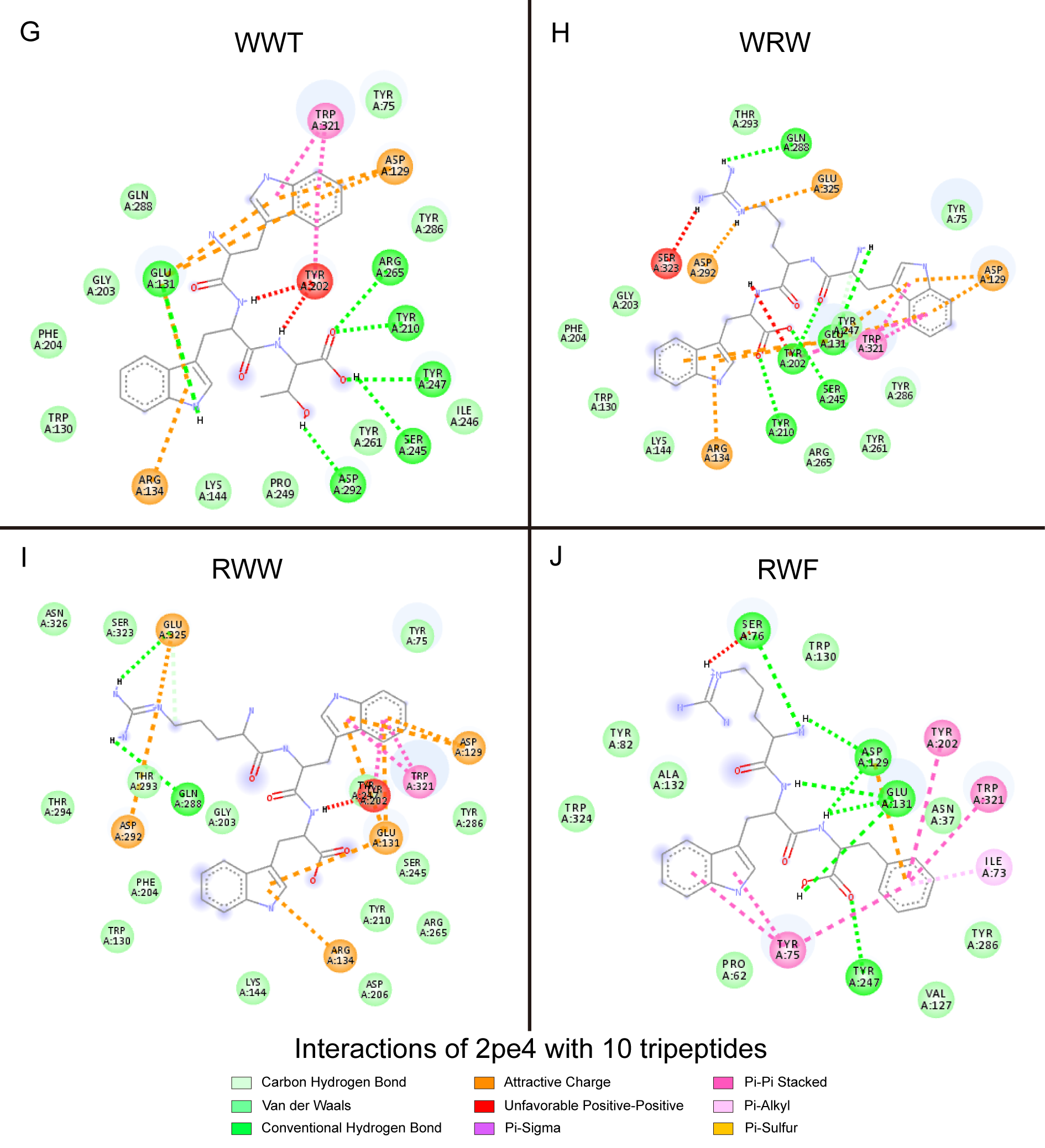


**Supplemental Figure 6.** The 2D dimensional visualization of molecular docking result based on the interaction between hyaluronidase (2pe4) and 10 tripeptides (HWW, WWH, HFW, FAW, FRW, TWW, WWT, WRW, RWW, RWF).


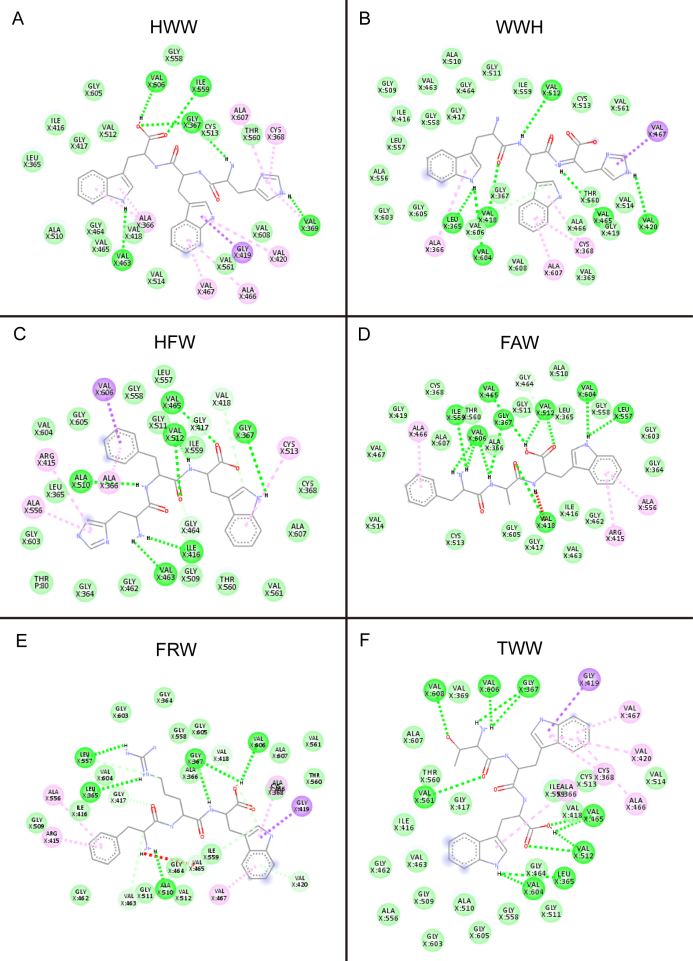

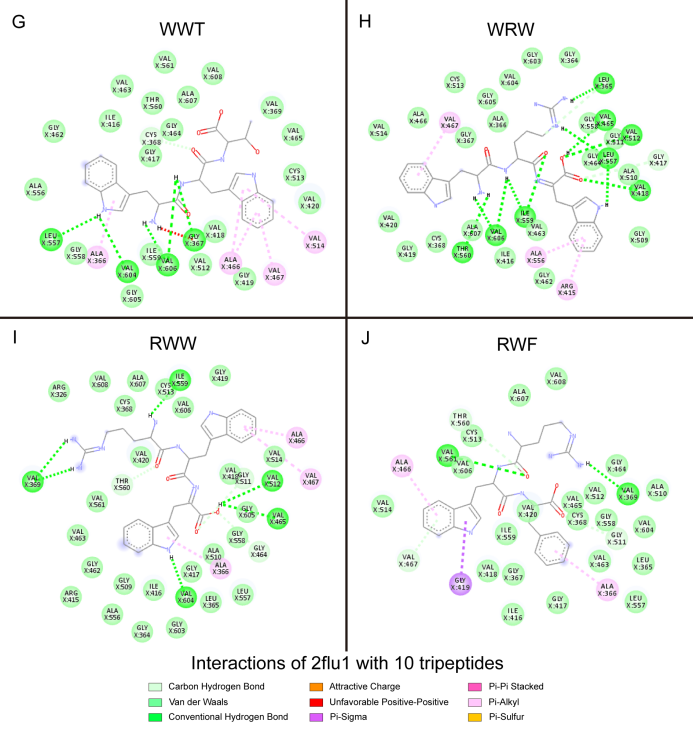


**Supplemental Figure 7.** The 2D dimensional visualization of molecular docking result based on the interaction between Keap1 (2flu1) and 10 tripeptides (HWW, WWH, HFW, FAW, FRW, TWW, WWT, WRW, RWW, RWF).


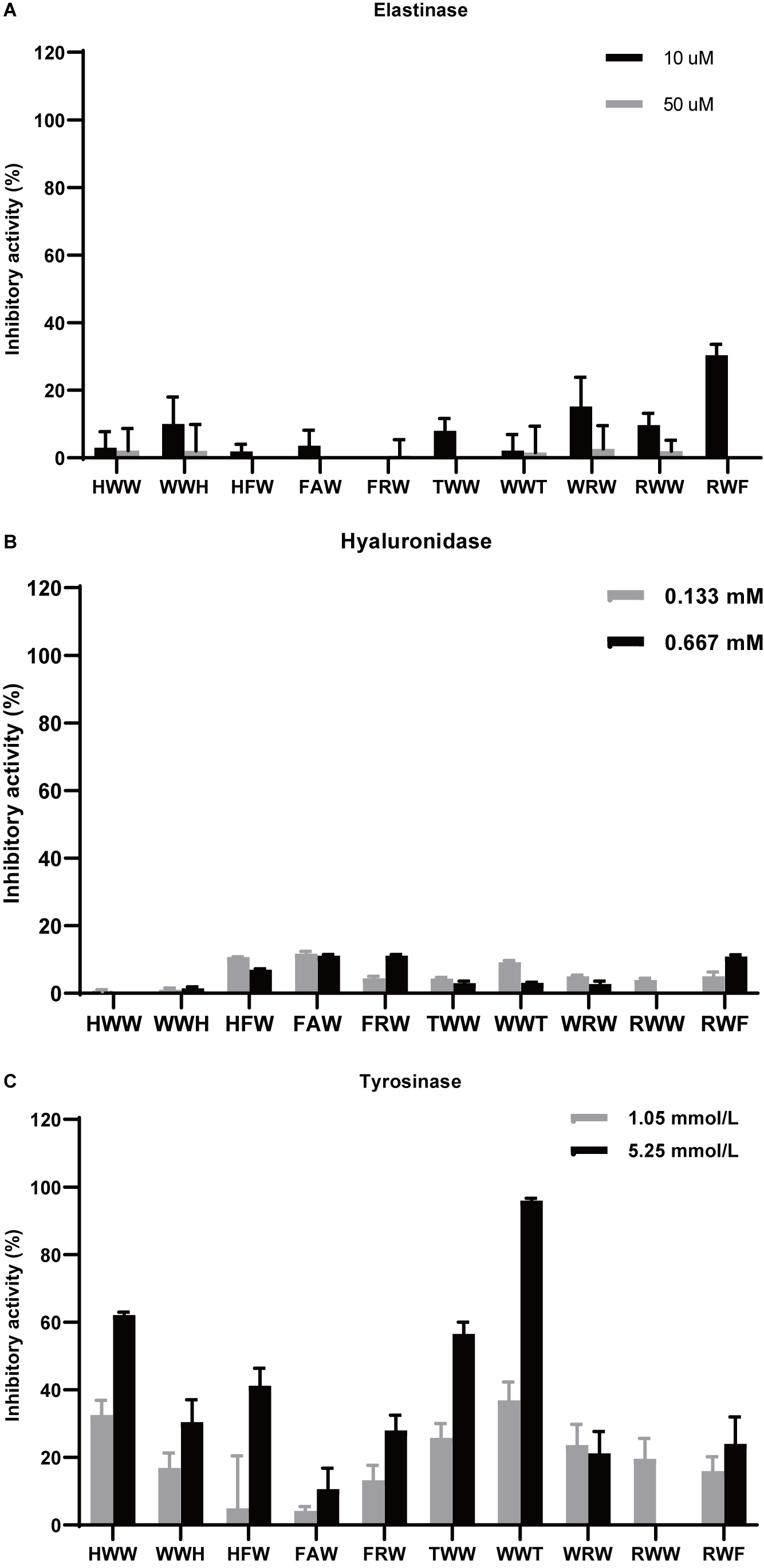


**Supplemental Figure 8.** Enzyme inhibitor activity detection of tripeptides. (A) Inhibitory activity of elastase; (B) Inhibitory activity of hyaluronidase; (C) Inhibitory activity of tyrosinase.


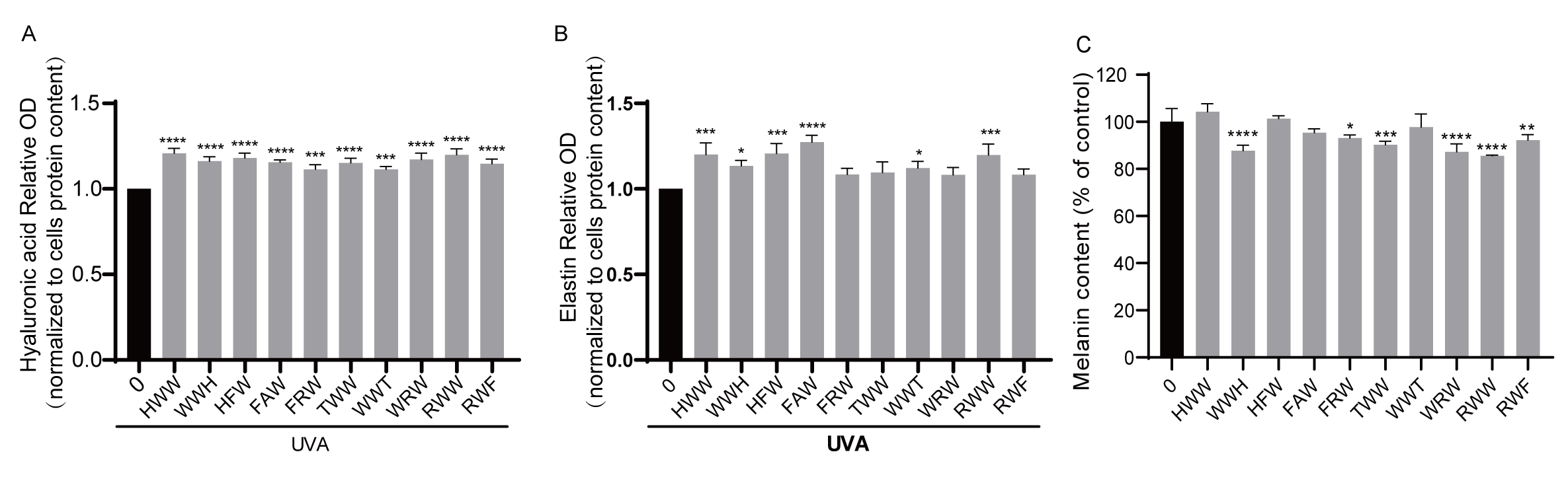


**Supplemental Figure 9.** The effect of designed tripeptides on the content changes of extracellular matrix components and melanin. (A) Hyaluronic acid level in the culture medium of HSF cell under condition of UVA irradiation. (B) Elastin level in the culture medium of HSF cell under condition of UVA irradiation; (C) Melanin content in the B16–F10 cells. * *P* ＜ 0.05，** *P* ＜ 0.01，*** *P* ＜ 0.001, **** *P* ＜ 0.0001.


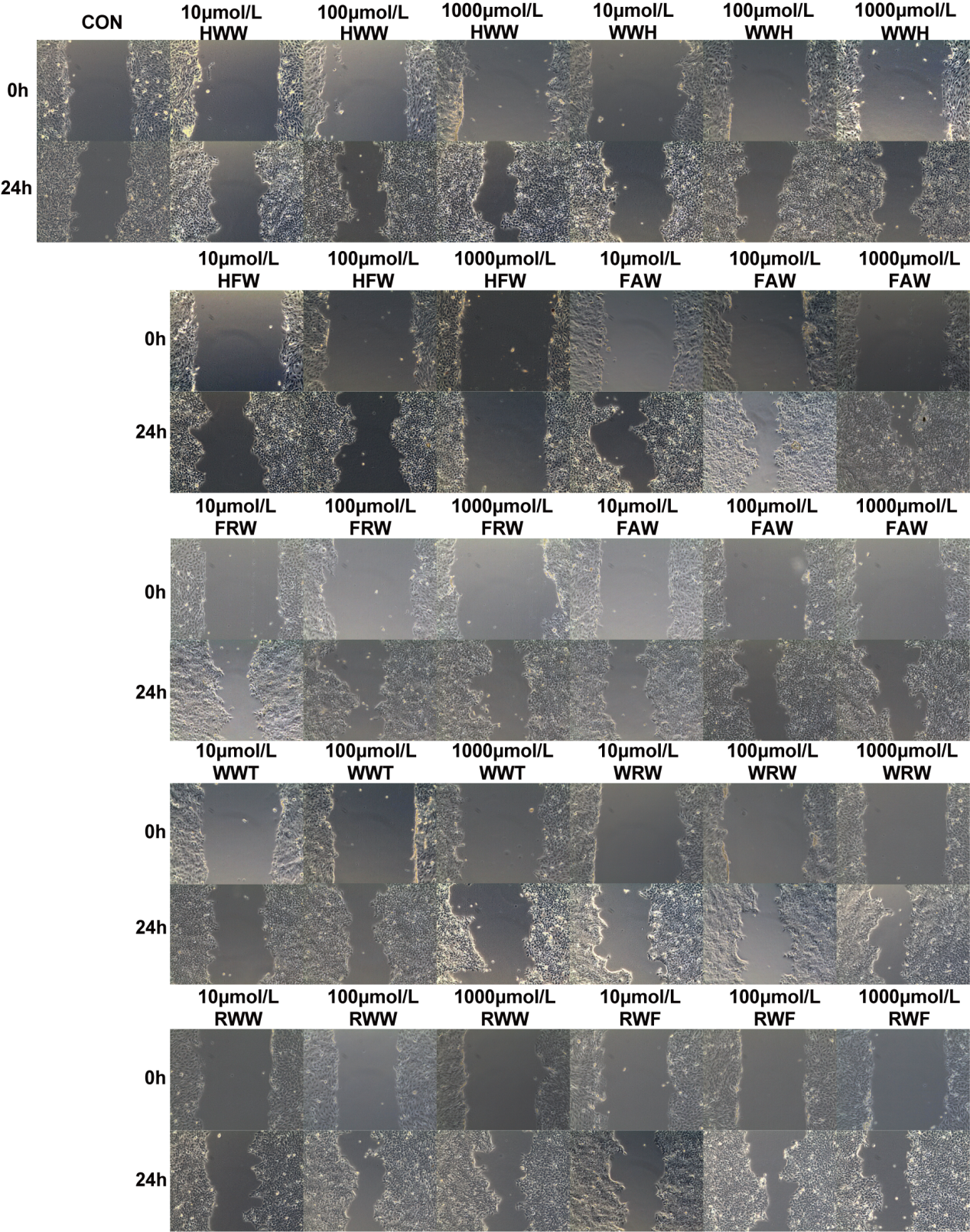


**Supplemental Figure 10.** Visualization results of HaCaT cell migration ability affected by ten tripeptides.
